# Increased body mass index is linked to systemic inflammation through altered chromatin co-accessibility in human preadipocytes

**DOI:** 10.1038/s41467-023-39919-y

**Published:** 2023-07-14

**Authors:** Kristina M. Garske, Asha Kar, Caroline Comenho, Brunilda Balliu, David Z. Pan, Yash V. Bhagat, Gregory Rosenberg, Amogha Koka, Sankha Subhra Das, Zong Miao, Janet S. Sinsheimer, Jaakko Kaprio, Kirsi H. Pietiläinen, Päivi Pajukanta

**Affiliations:** 1 Department of Human Genetics, David Geffen School of Medicine at UCLA, Los Angeles, CA 90095 USA; 2 Department of Computational Medicine, UCLA, Los Angeles, CA 90095 USA; 3 Bioinformatics Interdepartmental Program, UCLA, Los Angeles, CA 90095 USA; 4 Institute for Molecular Medicine Finland (FIMM), University of Helsinki, Helsinki, 00014 Finland; 5 Obesity Research Unit, Research Program for Clinical and Molecular Metabolism, Faculty of Medicine, University of Helsinki, Helsinki, 00014 Finland; 6 Obesity Center, Abdominal Center, Helsinki University Hospital and University of Helsinki, Helsinki, 00014 Finland; 7 Institute for Precision Heath, David Geffen School of Medicine at UCLA, Los Angeles, CA 90095 USA

**Keywords:** Obesity, Epigenomics, Gene expression

## Abstract

Obesity-induced adipose tissue dysfunction can cause low-grade inflammation and downstream obesity comorbidities. Although preadipocytes may contribute to this pro-inflammatory environment, the underlying mechanisms are unclear. We used human primary preadipocytes from body mass index (BMI) -discordant monozygotic (MZ) twin pairs to generate epigenetic (ATAC-sequence) and transcriptomic (RNA-sequence) data for testing whether increased BMI alters the subnuclear compartmentalization of open chromatin in the twins’ preadipocytes, causing downstream inflammation. Here we show that the co-accessibility of open chromatin, i.e. compartmentalization of chromatin activity, is altered in the higher vs lower BMI MZ siblings for a large subset ( ~ 88.5 Mb) of the active subnuclear compartments. Using the UK Biobank we show that variants within these regions contribute to systemic inflammation through interactions with BMI on C-reactive protein. In summary, open chromatin co-accessibility in human preadipocytes is disrupted among the higher BMI siblings, suggesting a mechanism how obesity may lead to inflammation via gene-environment interactions.

## Abstract (web summary)

Preadipocytes contribute to the pro-inflammatory environment in obesity, via unknown mechanisms. Here, comparing monozygotic twin pairs, the authors show that co-accessibility of chromatin in preadipocytes is altered in siblings with higher compared to lower BMI, and that variants in these regions contribute to systemic inflammation via interactions with BMI.

## Introduction

The obesity epidemic is driving concomitant alarming increases in obesity comorbidities, such as type 2 diabetes (T2D), coronary artery disease (CAD), and nonalcoholic fatty liver disease (NAFLD)^1–3^. Obesity is also one of the key risk factors for severe COVID-19 outcomes^4,5^, most likely not only due to the altered mechanics of lung ventilation but also due to the low-grade inflammation induced by obesity^6^. Recent assessment of polygenic risk scores (PRSs) for the obesity surrogate trait, body mass index (BMI), highlights how accumulation of risk variants is associated with the level of BMI and higher odds of having obesity comorbidities, T2D or CAD, in the UK Biobank (UKB)^7^. However, not all individuals with obesity exhibit metabolic profiles associated with poor cardiometabolic health outcomes, and this variation can be due to genetics, environmental factors, and interactions between them^7,8^. Therefore, understanding the pathophysiological mechanisms in obesity that contribute to metabolically and immunologically unhealthy phenotypes can improve risk assessment of genetic and environmental contributions to these clinically important traits. This will ultimately inform treatment strategies to decrease morbidity and mortality due to the obesity epidemic.

Chronic low-grade inflammation is a hallmark of obesity that contributes to the development of obesity comorbidities, such as insulin resistance and atherosclerosis^1^. In addition to the known role of adipose macrophages in this process^1,9^, there is increasing evidence for the role of preadipocytes in the development of dysfunctional adipose tissue and the pro-inflammatory state seen in obesity^10^. Preadipocytes are capable of secreting pro-inflammatory cytokines at appreciable levels, and can exhibit gene expression profiles similar to macrophages^10,11^. However, this has been predominantly shown in vitro, in response to treating preadipocytes with media containing pro-inflammatory molecules^12–15^. The underlying biological mechanisms through which preadipocytes contribute to the pro-inflammatory signals within the adipose tissue of individuals with obesity remain unclear.

We hypothesized that the obesogenic environment disrupts the proper function of preadipocytes through altered co-accessibility of open chromatin, the identification of which may pinpoint mechanisms driving obesity-related inflammation. To discover novel epigenetic co-accessibility changes that would link increased BMI to systemic inflammation in the key adipose cell-type, human primary preadipocytes, we investigated monozygotic (MZ) twins discordant for BMI (ΔBMI ≥3 kg/m^2^), and thus, in each MZ twin pair, the lower-BMI twin provides the control individual and the higher-BMI twin provides the case individual, controlled for the same genetic variants. This design allows us to separate observed epigenetic changes from the effects of genetic variants, given that the MZ twin pairs have a different BMI but the same genome. Thus, our unique BMI-discordant MZ twin design provides individual-level genetic control to our study, rather than the currently used BMI cut points for lean and obese that are based on health-related outcomes at the population level. Therefore, any differences we observe between these MZ twin siblings discordant for BMI will likely be due to environmental differences in the individual’s history.

We first generated an assay for transposase-accessible chromatin (ATAC) -sequencing and RNA-seq data in the twins’ preadipocytes, and promoter Capture Hi-C (pCHi-C) data in an independent source of human primary preadipocytes from one individual^16^. By integrating these data and leveraging co-accessibility information across all preadipocyte samples from the twins, we identified subnuclear compartments of chromatin activity, previously defined as active (A) and inactive (B) compartments^17^. The active A compartments exhibited significant differences in compartmentalization of chromatin activity, defined by the A compartment level of co-accessibility, between the lower and higher BMI MZ sibling groups. We show that these active A compartment regions with altered open chromatin co-accessibility may contribute to inflammation through gene–BMI interactions in the UK Biobank (UKB), indicating that we have identified preadipocyte-origin genomic mechanisms underlying the pro-inflammatory environment seen in obesity.

## Results

### Identification of A/B compartments in human primary preadipocytes

We have an ongoing collection of a unique, deeply phenotyped cohort of 50 Finnish monozygotic (MZ) twin pairs who are discordant for BMI (ΔBMI ≥3 kg/m^2^)^18,19^ (see Methods). The phenotypic and metabolic characteristics of these twins are summarized in Supplementary Data 1. A paired *t*-test of phenotypic measurements indicates that many traits are significantly different between the lower and higher BMI groups of siblings, including C-reactive protein (CRP), which is a measure of systemic inflammation, important comorbidity of obesity (Supplementary Data 1). We hypothesized that there are changes in the co-accessibility of open chromatin in the preadipocytes (PAd) under conditions of increased BMI, which could lead to adipose tissue dysregulation and contribute to the difference in inflammatory profiles. We, therefore, isolated the primary subcutaneous PAd from the subcutaneous adipose biopsies of 10 of the BMI-discordant MZ pairs (*n* = 20) (see Methods). The phenotypic characteristics of this subset of pairs for whom we collected the PAd are summarized separately in Supplementary Data 2.

**Fig. 1:**
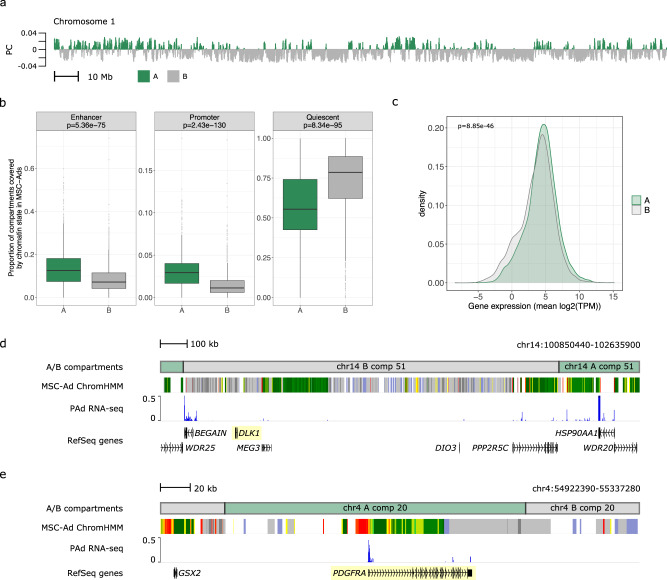
A/B compartment identification using ATAC-seq co-accessibility in human primary preadipocytes. **a** PAd A/B compartments on chromosome 1. Each bar represents a 100-kb bin and the height of the bar is the projection onto the first eigenvector of the 100-kb bin co-accessibility matrix across chromosome 1. The sign switches at A/B compartment boundaries; positive values correspond to A compartments (green) and negative values correspond to B compartments (gray). **b** Coverage of enhancer, promoter, and quiescent ChromHMM chromatin states in the A (*n* = 1551 compartments) and B (*n* = 1557 compartments) compartments. *P* values correspond to the two-sided Wilcoxon rank-sum test comparing the A compartment to B compartment coverage for each chromatin state. Boxplot center represents the median coverage of the indicated chromatin state in the compartment type, the upper and lower bounds of the box represent the 75th and 25th percentile, respectively, and the upper and lower whiskers represent the highest (non-outlier) and lowest (non-outlier) values, respectively. **c** Density distribution of the gene expression (mean log_2_(TPM)) in the A (green) and B (gray) compartments shows higher expression in the A compartments. *P* value corresponds to the two-sided Wilcoxon rank-sum test comparing the gene expression in the A (*n* = 6554 expressed genes) compartments to the gene expression in the B (*n* = 5708 expressed genes) compartments. Genome browser snapshots of two preadipocyte marker genes: **d** *DLK1*, an early preadipocyte marker, is located within a B compartment on chromosome 4 and is not expressed; and **e** *PDGFRA*, a late preadipocyte marker, is located within an A compartment on chromosome 14 and is expressed. The ChromHMM state track is directly from Roadmap Epigenomics on the WashU Epigenome Browser. PC indicates principal components; MSC-Ad mesenchymal stem cell-derived adipocyte cultured cells, and PAd preadipocyte. Source data are provided as a Source Data file.

To characterize the PAd at the level of subnuclear compartmentalization of chromatin activity, we performed ATAC-seq on the BMI-discordant MZ twin pairs’ PAd. For all downstream analyses, we report the findings from the nine pairs for whom the ATAC-seq passed quality control (see Methods; Supplementary Data 3; Supplementary Table 1; and Supplementary Figs. 1, 2a–d). We inferred A and B genomic compartments, which are broadly associated with active or inactive regions of genome^17^, respectively, using the co-accessibility information from the ATAC-seq coverage across 100-kb bins, as described previously^20^ (see Methods) (Fig. 1a; Supplementary Data 4; and Supplementary Fig. 2e–h). In the MZ twin PAd, the A compartments were shorter than the B compartments, with a median of 300 kb compared with 600 kb in the B compartments (*p*_Wilcoxon_ = 3.08 × 10^−76^) (Supplementary Fig. 3a). B compartments made up an average of 74.8 ± 9.7% (s.d.) of each chromosome (Supplementary Fig. 3b). We validated the compartment detection by characterizing the stratification of known functional features of chromatin compartmentalization across the A and B compartments^17,21^. The proportion of chromosomes making up B compartments is significantly correlated (Spearman’s rho = 0.68, *p* = 6.8 × 10^−4^) with the percent of the chromosome that is comprised of gene deserts (Supplementary Fig. 3c). As previously reported^17,21^, gene deserts were largely restricted to B compartments (Supplementary Fig. 3d) and the gene density in the A compartments was significantly higher than in the B compartments (*p*_Wilcoxon_ = 3.34 × 10^−69^) (Supplementary Fig. 3e).

### Preadipocyte A/B compartments reflect cell-type-specific gene regulatory activity

To validate our A/B compartment detection with external datasets and address the cell-type-specificity of gene regulatory activity in the PAd A/B compartments, we compared their coverage of chromatin states across 127 ENCODE cell types using the imputed 25-state model from ChromHMM^22^. We found that the A compartment coverage of enhancer chromatin states was highest for the mesenchymal stem cell-derived adipocyte cultured cells (MSC-Ad) *(p*_adj_ < 0.05), and next highest in adipose-derived mesenchymal stem cell cultured cells (Ad-MSC), in line with the primary PAd being at a developmental stage similar to these two cell types (Supplementary Fig. 4). There was a similar trend for the A compartment coverage of promoter chromatin states for MSC-Ad (Supplementary Fig. 4). Conversely, the A compartment coverage of quiescent chromatin states was significantly lower in the MSC-Ad than all other cell types (*p*_adj_ < 0.05) except Ad-MSCs and primary breast myoepithelial cells (Supplementary Fig. 4). Since the A compartment coverage was most specific for MSC-Ad, we consider the primary PAd to be most similar to this ENCODE cell type. We, therefore, used these chromatin states for all subsequent analyses using ChromHMM.

To confirm that measures of active gene regulation are more restricted to A compartments than B compartments, we assessed the coverage of active or inactive chromatin states within the A/B compartments. The A compartment coverage was higher than the B compartment coverage for the enhancer (*p*_Wilcoxon_ = 5.36 × 10^−75^) and promoter (*p*_Wilcoxon_ = 2.43 × 10^−130^) chromatin states, whereas the B compartment coverage was higher than the A compartment coverage for quiescent states (*p*_Wilcoxon_ = 8.34 × 10^−95^) (Fig. 1b), in line with active gene regulation being more prevalent in the A compartments. Taken together, we used human primary PAd ATAC-seq data to infer A/B compartments that can be used to better understand the co-accessibility of open chromatin of these cells.

To determine whether the compartmentalization of active (A) and inactive (B) chromatin states associates with higher and lower gene expression, respectively, we performed RNA-seq on the PAd from the ten BMI-discordant MZ pairs (*n* = 20) (see Methods; Supplementary Data 2; Supplementary Table 2; and Supplementary Fig. 5). Genes in the A compartments have higher mean expression than genes in the B compartments (*p*_Wilcoxon_ = 8.85 × 10^−46^), in line with the fact that A compartments are enriched for molecular signatures of active gene regulation (Fig. 1c). A clear example of how the A/B compartments define cell-type-specific genomic programming is presented in Fig. 1d, e. Consistent with these primary cells being at a later developmental time point^23^, the early PAd marker, *DLK1*, is located within a B compartment and has negligible expression in the preadipocytes (Fig. 1d). Conversely, the later PAd marker, *PDGFRA*, is located within an A compartment and is clearly expressed (Fig. 1e). Furthermore, the well-established adipocyte-specific adipocytokine, *ADIPOQ*, is located within a PAd B compartment, in line with this gene not being expressed until the later stages of adipocyte differentiation (Supplementary Data 4). Taken together, the inference of the A and B compartments is in accordance with previously published hallmark features of subnuclear compartments.

### Promoter–enhancer interactions are enriched in the A compartments

We next aimed to determine whether the A/B compartments demarcate regions that have been shown to be physically interacting in an independent source of human primary PAd. To link regulatory elements to their target promoters through chromosomal interactions^24^, we identified promoter interactions in human primary PAd using our existing promoter Capture Hi-C (pCHi-C) data from a European origin individual^16^ (Supplementary Table 3). To assess whether the PAd pCHi-C interactions correspond to the subnuclear A/B compartmentalization, we examined whether the two ends of the pCHi-C interactions land in the same or different compartments. For 51,974 of the 76,473 PAd interactions (68.0%), both ends landed in the same compartment, with 25,686 of these (49.4%) being contained within the same A compartment, and 26,288 (50.6%) of these interactions being in the same B compartment. Given that the B compartments make up ~75% of the genome in the preadipocytes (Supplementary Fig. 3b), this suggests that the pCHi-C interactions are enriched within the A compartments. To determine whether the proportion of pCHi-C interactions in the A compartments is higher than expected by chance alone, we permuted the compartment locations and re-calculated how often both ends of the pCHi-C interactions land in the same A compartment (see Methods). The proportion of interactions that have both ends landing in the same permuted A compartments is, on average, 18.5 ± 1.1% (s.d.), meaning that there is a 2.67-fold enrichment of pCHi-C interactions in the A compartments (*p* < 1 × 10^−04^). This is in line with the pCHi-C interactions being regulatory and thus being more prevalent in A compartment regions of active gene regulation. It has previously been reported that genes involved in pCHi-C interactions are more highly expressed^25^. We found that this is only true for genes located within the A compartments (*p*_wilcoxon_ = 1.38 × 10^−09^), whereas this was not the case for genes in the B compartments (*p*_wilcoxon_ = 0.873) (Supplementary Fig. 6).

Taken together, our data suggest that PAd pCHi-C interactions are enriched in the PAd A compartments, and that the previously reported higher expression of genes involved in pCHi-C interactions may be dependent upon the gene landing in A, rather than B compartments (Supplementary Fig. 6). We chose to focus on the A compartments for the remainder of our study, given the evidence that active gene regulation is occurring in these regions, and thus they are likely important for PAd function.

### Genome-wide A compartment co-accessibility is decreased in MZ siblings with a higher BMI

**Fig. 2:**
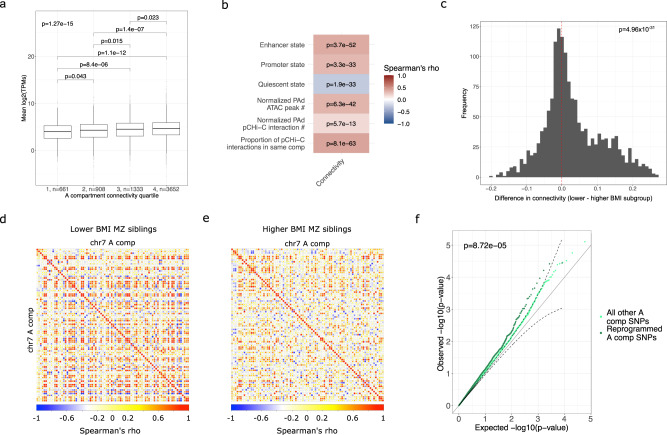
The A compartment co-accessibility differs between the lower and higher BMI twins and contributes to genotype-by-BMI interactions affecting CRP in the UK Biobank. **a** Boxplots show the mean expression of genes in the A compartments, stratified into quartiles of the A compartment connectivity. The number of expressed genes in each quartile is noted. The overall *p* value refers to the Kruskal–Wallis two-sided test for expression differences across the A compartment connectivity quartiles. Pairwise comparisons are from the post hoc Dunn test after correction for multiple testing using the Holm procedure. Boxplot center represents the median expression of the indicated gene set in the indicated compartment type, the upper and lower bounds of the box represent the 75th and 25th percentile, respectively, and the upper and lower whiskers represent the highest (non-outlier) and lowest (non-outlier) values, respectively. **b** Heatmap shows the correlation of the A compartment connectivity with ChromHMM chromatin states and this study’s PAd ATAC-seq and pCHi-C data. *P* values correspond to the significance of Spearman’s rank correlation FDR. **c** Histogram of the differences in the A compartment connectivity between the lower and higher BMI MZ twins. The red dashed line at x = 0 denotes the null hypothesis that there are not genome-wide co-accessibility differences between the twins. The *p* value corresponds to the one-sample, two-sided Wilcoxon test for the co-accessibility differences. The lower BMI twins exhibit higher A compartment connectivity compared to the higher BMI twin siblings. Correlation plots show that the co-accessibility of the A compartments on chromosome 7 is stronger in **d** the lower BMI MZ siblings relative to **e** the higher BMI MZ siblings. This is a representative image from all 22 autosomal chromosomes. **f** Q-Q plots for the uniform distribution of the *p* values for genotype-by-BMI interaction effects on CRP in the UKB, stratified by whether SNPs land in the A compartments with altered co-accessibility or not. Confidence intervals (dashed lines) were calculated for the altered A compartment *p* values. The *p* value corresponds to the two-sided Wilcoxon rank-sum test for differences in the *p* value distribution between the altered and unaltered A compartments. Source data are provided as a Source Data file.

We hypothesized that the BMI-discordant MZ pairs would exhibit differences in the compartmentalization of chromatin activity in their preadipocytes. To establish a measure of compartmentalization to compare the twins, we calculated the level of co-accessibility, i.e., the degree of correlation between a given A compartment and all other A compartments (see Methods), for each A compartment individually. We tested whether this co-accessibility measure is associated with gene expression and accumulation of active chromatin states, following the hypothesis that the A compartment co-accessibility reflects active and coordinated gene regulation between loci. We found that the A compartment co-accessibility is significantly associated with gene expression (*p*_KW_ = 1.27 × 10^−15^) (Fig. 2a), and all measures of active gene regulation based on chromatin state, PAd pCHi-C interactions, and PAd accessible chromatin (Fig. 2b). Thus, the A compartment degree of co-accessibility can be used as a metric that captures various levels of gene regulation associated with it.

To assess whether the genome-wide A compartment co-accessibility is different between the BMI-discordant MZ siblings, we separated the lower and higher BMI siblings (*n* = 9 MZ pairs split into lower and higher BMI subgroups) and re-computed the level of co-accessibility of the A compartments in each set separately (see Methods). We found that the A compartment co-accessibility measures are significantly higher in the lower BMI set of siblings than in the higher BMI siblings (*p* = 4.96 × 10^−31^) (Fig. 2c–e). This overall decline of A compartment co-accessibility in the higher BMI siblings suggests that the PAd compartmentalization of chromatin activity is altered when comparing individuals with a higher BMI to those with a lower BMI.

To identify specific A compartments that exhibit the strongest co-accessibility differences between the lower and higher BMI MZ twin siblings, we permuted the lower and higher BMI labels between siblings (*n* = 2^9 pairs^ or 512 permutations) and re-calculated the difference in the degree of co-accessibility between the lower and higher BMI subgroups of siblings for all compartments (see Methods). We defined the A compartments with a permutation *p* value of less than 0.01, totaling ~88.5 Mb, as those with altered co-accessibility in response to BMI in the BMI-discordant MZ pairs (Supplementary Data 4). These altered A compartments thus represent regions that are mechanistic candidates for gene–environment interactions (GxEs) originating in human PAd.

### PAd A compartments with altered co-accessibility are enriched for the heritability of CRP and contribute to gene–BMI interaction effects on CRP in the UK Biobank

We first tested whether there is evidence that variants that land in genomic regions that are active in PAd contribute to marginal genetic effects on systemic inflammation. We used partitioned LD-score (LDSC) regression^26,27^ to partition the heritability of CRP into the PAd A and B compartments, while also stratifying the A compartments into those with altered co-accessibility in the BMI-discordant MZ twins and those with unaltered co-accessibility in the twins (see Methods). The B compartments were significantly depleted for the heritability of CRP (enrichment = 0.840, *p* = 1.99 × 10^−09^), whereas the A compartments (enrichment = 1.33, *p* = 7.05 × 10^−04^) and the A compartments with altered co-accessibility (enrichment = 3.10, *p* = 6.19 × 10^−03^) were both significantly enriched for the heritability of CRP (Table 1). We also tested whether the heritability of BMI and other obesity-related traits, including serum triglycerides (TGs), alanine aminotransferase (ALT), blood glucose levels, LDL and total cholesterol, systolic blood pressure (BP), and forced vital capacity (FVC), are enriched in the PAd A compartments. All cardiometabolic traits tested exhibited a significant depletion of heritability in the B compartments (enrichment = 0.752–0.946, FDR <0.05). Interestingly, the A compartments were significantly enriched for the heritability of all obesity-related traits (enrichment = 1.33-1.72, FDR <0.05), but not BMI itself, in line with the previously identified enrichment of brain mechanisms in BMI GWAS loci^28,29^.

**Table 1:** Partitioned LDSC analysis shows that the A compartments are significantly enriched for the proportion of CRP heritability while the B compartments are depleted

PAd A/B compartment category	Prop. of SNPs	Prop. of *h*^*2*^	Prop. of *h*^*2*^ SE	Enrichment	Enrichment SE	Enrichment *p* value
B	0.773	0.650	0.017	0.840	0.022	1.99 × 10^−09^
A (unaltered in MZ twins)	0.199	0.265	0.020	1.33	0.099	7.05 × 10^−04^
A (altered in MZ twins)	0.0276	0.0856	0.020	3.10	0.72	6.19 × 10^−03^

SE indicates standard error, prop. proportion, and *h*^*2*^ heritability. Partitioned LD-score regression (LDSC)^26,27^ was performed using the C-reactive protein (CRP) summary statistics from the UK Biobank round 2 GWAS results from 343,524 individuals, hosted at the Neale Lab website (http://www.nealelab.is/uk-biobank/). Heritability was partitioned using the SNPs in the identified PAd B and A compartments, the latter of which was further stratified into the PAd A compartments with unaltered open chromatin co-accessibility, or altered co-accessibility in the higher BMI twin siblings from the BMI-discordant MZ twins. The SE for the proportion of *h*^*2*^ and enrichment were calculated from the block jackknife resampling using the LDSC method. The *p* value is calculated using the proportion of heritability and the proportion of heritability SE from the block jackknife resampling, and computing a *z*-score (two-sided test for significance of enrichment). The B compartments exhibit a significant depletion in the proportion of heritability explained, similar to repressed regions of the genome^26^; whereas the A compartments are significantly enriched for the proportion of CRP heritability explained in these regions.

To next determine whether the BMI-responsive, altered A compartments identified in the BMI-discordant MZ twin pairs’ PAd are more likely to harbor GxE effects on inflammation, we performed a GxE scan in the UKB, testing all SNPs in the A compartments for the effect of SNPs interacting with BMI on CRP levels (see Methods). We compared the distribution of the GxE *p* values in the A compartments with altered co-accessibility of open chromatin (88.5 Mb) to all other A compartments (561 Mb) (Supplementary Data 4). Indeed, we found that the altered compartments have a higher accumulation of low *p* value GxE signals than all other A compartments (*p*_Wilcoxon_ = 8.72 × 10^−05^) (Fig. 2f). This suggests that the regions exhibiting BMI-dependent PAd co-accessibility differences identified in the BMI-discordant MZ pairs likely to harbor many small-effect GxEs affecting inflammation in humans, above what is seen in the A compartments alone.

### Clustering of the A compartments identifies compartment subcommunities that are important for distinct preadipocyte functions

**Fig. 3:**
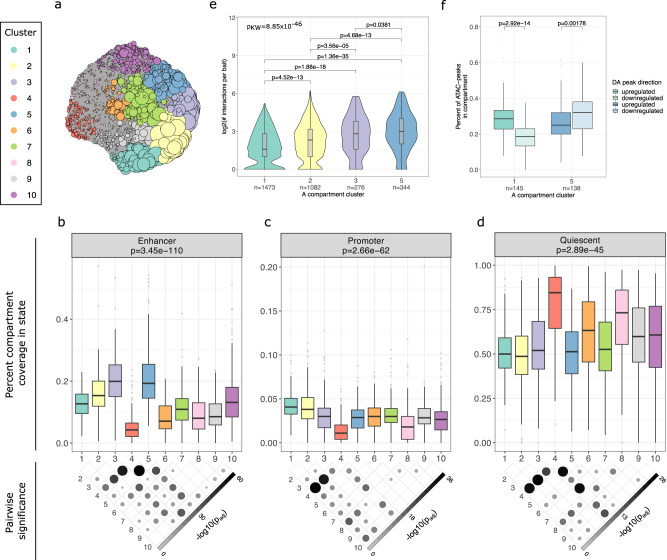
The A compartment clustering reveals differential accumulation of chromatin states and gene regulatory landscapes. **a** iGraph layout of the A compartment clusters after UMAP dimensionality reduction and Louvain clustering. Each circle represents an A compartment. Colors represent the ten clusters that were identified. The size of the circles is proportional to the level of co-accessibility of that A compartment, showing that clusters 1, 2, 3, and 5 have the highest levels of co-accessibility. The A compartment cluster coverage of enhancer (**b**), promoter (**c**), and quiescent (**d**) ChromHMM chromatin states are indicated with boxplots. Overall *p* values (top) correspond to the two-sided Kruskal–Wallis test comparing the coverage across the A compartment clusters. The *p* value map below the plot denotes which pairwise differences are significant (*p* < 0.05) in the post hoc Dunn test, after correcting for multiple testing using the Holm procedure. The number of compartments in each cluster is listed in Supplementary Table 4. **e** Violin plots with inlaid boxplots show the number of pCHi-C interactions per promoter in the four A compartment clusters. The overall *p* value corresponds to the two-sided Kruskal–Wallis test and the pairwise comparisons denote the *p* values from the post hoc Dunn test, after correcting for multiple testing using the Holm procedure. The violin plot shows the kernel probability density of the data. **f** Boxplots show the proportion of ATAC peaks in the cluster A compartments that are upregulated (higher accessibility in D1 relative to PAd) or downregulated (lower accessibility in D1 relative to PAd) after 24 h of PAd differentiation into adipocytes. The *p* values correspond to the two-sided paired Wilcoxon rank-sum test for differences between the proportion of up- or down-regulated peaks in each of the compartment clusters separately. See also Supplementary Figs. 7, 8, Supplementary Data 5, 6, and Supplementary Table 4. For all boxplots, the center represents the median gene density of the compartment type, the upper and lower bounds of the box represent the 75th and 25th percentile, respectively, and the upper and lower whiskers represent the highest (non-outlier) and lowest (non-outlier) values, respectively. Source data are provided as a Source Data file.

To gain insight into the genomic regulatory mechanisms that contribute to the regionally enriched candidate GxEs affecting inflammation in the UKB, we clustered the A compartments after UMAP dimensionality reduction^30^ to 2 variance components (see Methods). This clustering approach identified ten clusters that exhibit varying levels of co-accessibility, containing between 107 and 230 A compartments (Fig. 3a; Supplementary Fig. 7a; Supplementary Data 4; and Supplementary Table 4). The total sum of the lengths (in Mbs) of the compartments in each of the A compartment clusters is given in Supplementary Table 4. As expected, given the observed correlations between co-accessibility and chromatin states (Fig. 2b), the clusters containing the A compartments with the highest levels of co-accessibility (clusters 1, 2, 3, and 5; *p*_adj_ < 0.05) (Fig. 3a and Supplementary Fig. 7a) also exhibit the highest coverage of enhancer (Fig. 3b) (clusters 2, 3, and 5; *p*_adj_ < 0.05) or promoter (Fig. 3c) (clusters 1 and 2; *p*_adj_ < 0.05) chromatin states; and have the lowest coverage of quiescent chromatin states (*p*_adj_ < 0.05) (Fig. 3d). Thus, as clusters 1, 2, 3, and 5 likely represent the most important A compartments for PAd co-accessibility and function, we chose to focus on these clusters for the remainder of the study.

The differences in enhancer and promoter chromatin state coverage between the four main A compartment clusters 1, 2, 3 and 5 suggest that there may be differential gene regulatory mechanisms functioning within each cluster. In support of this, we found that cluster 5, which exhibits the highest enhancer chromatin state coverage (Fig. 3b), is enriched 3.2-fold for PAd super-enhancers (FDR_hypergeom_ = 7.8 × 10^−04^) (see Methods). Cluster 5 also exhibits the highest number of PAd pCHi-C interactions per promoter (*p*_KW_ = 8.85 × 10^−46^), particularly when compared with cluster 1 (mean of eight interactions per promoter in cluster 5 versus the mean of three interactions per promoter in cluster 1) (Fig. 3e). Accordingly, the gene expression in cluster 1 is significantly lower than in clusters 2, 3, and 5 (*p*_KW_ = 2.50 × 10^−08^) (Supplementary Fig. 7b). These data suggest that in contrast to the highly interacting, enhancer-enriched cluster 5, cluster 1 may be more developmentally primed.

One feature that has been previously reported to be more common in cells that are primed for differentiation is a higher number of promoter-promoter (P-P) interactions in pCHi-C data^31^. We found that cluster 1 does, in fact, exhibit a higher proportion of P-P interactions relative to clusters 2, 3, and 5 (*p*_KW_ = 1.05 × 10^−24^) (Supplementary Fig. 7c). We performed a gene ontology (GO) enrichment analysis on the genes in each of the clusters separately (Supplementary Fig. 8 and Supplementary Data 5) (see Methods). Importantly, performing enrichment analyses on genes selected from large genomic regions can lead to spurious enrichments due to clusters of gene families or genes with similar functions^32^. We, therefore, used the Network Enrichment Analysis Tool (NEAT)^33^, which uses information about the relationship between genes (e.g., genes in the same co-expression network) to test for functional enrichment. To provide the network information to NEAT, we performed weighted gene co-expression network analysis (WGCNA)^34^ using the RNA-seq data from all of the A compartment genes together (Supplementary Data 6). Genes from each A compartment cluster were assigned a co-expression module, and this network information was provided to NEAT for the A compartment GO enrichment. We found that cluster 1 is enriched for developmental processes, cell polarity, and cell adhesion, in line with this cluster being important for cellular priming^35^ (Supplementary Fig. 8 and Supplementary Data 5). Notably, immune-related processes such as leukocyte chemotaxis and proliferation, response to cytokine, and apoptotic cell clearance are also enriched in the A compartment cluster 1 (Supplementary Fig. 8 and Supplementary Data 5).

Next, we performed a transcription factor (TF) motif enrichment analysis using HOMER^36^ (see Methods) on each of the clusters separately and identified 20 motif sequences (matching 18 distinct TFs) that are significantly enriched in the A compartment cluster 1, relative to all other A compartment clusters (Supplementary Table 5). One of these motifs matches the sequence for the Sterol Regulatory Element-Binding Protein 1 (SREBP1), which is a master transcriptional activator required for lipid homeostasis and is involved in adipogenesis^37,38^, supporting our conclusion that the A compartment cluster 1 regions are developmentally primed PAd elements.

To further examine whether the regions in the A compartment cluster 1 are likely to be developmentally primed relative to the A compartment cluster 5, we assessed the effects of initiating the MZ twin pairs’ PAd differentiation into adipocytes for 24 h by performing ATAC-seq on the cells at this developmental time point (see Methods; Supplementary Table 1 and Supplementary Figs. 1, 9). The A compartment cluster 5, which has the highest accumulation of enhancer chromatin state coverage, exhibited a higher proportion of ATAC-seq peaks with decreased accessibility after the first 24 h of differentiation (Fig. 3f). This is consistent with cluster 5 being made up of genomic regions that are specifically important for PAd function, in line with the strong enrichment of super-enhancers (see above). On the other hand, cluster 1 showed the opposite trend, with a higher proportion of ATAC-seq peaks being more accessible after the first 24 h of differentiation, relative to PAd (Fig. 3f). Taken together, the chromatin state and super-enhancer coverage, as well as the differential responses to early differentiation signals, suggest that the A compartment clusters represent regions of the genome that are functionally related and exhibit distinct gene regulatory mechanisms in PAd.

### The developmentally primed PAd A compartment cluster 1 is enriched for the heritability of CRP and obesity-related traits in the UK Biobank

**Fig. 4:**
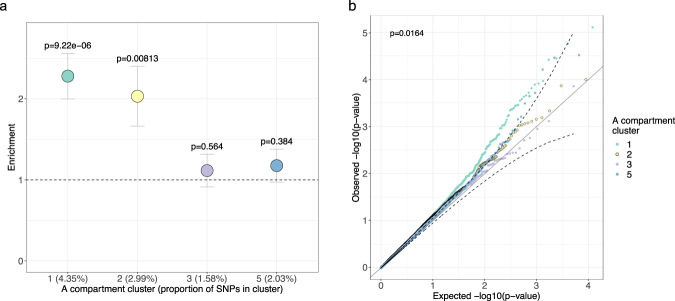
The A compartment cluster 1 contributes significantly to the heritability of CRP and is enriched for genotype-by-BMI interaction effects on CRP in the UK Biobank. **a** Dotplot shows the enrichment of heritability for CRP in the different A compartment clusters relative to the null hypothesis of the uniform contribution from all SNPs. Partitioned LD-score regression (LDSC)^26,27^ was performed using the C-reactive protein (CRP) summary statistics from the UK Biobank round 2 GWAS results from 343,524 individuals, hosted at the Neale Lab website (http://www.nealelab.is/uk-biobank/). Error bars represent the heritability enrichment standard error (SE) and the data were presented as the enrichment of heritability for CRP calculated from LDSC ± the enrichment SE. The SE for the proportion of *h*^*2*^ and enrichment were calculated from the block jackknife resampling using the LDSC method. The *p* value is calculated using the proportion of heritability and the proportion of heritability SE from the block jackknife resampling, and computing a *z*-score (two-sided test for significance of enrichment). The x-axis tick marks list the A compartment cluster with the proportion of SNPs in that cluster in parentheses. **b** Q-Q plots for the uniform distribution of the *p* values for the genotype-by-BMI interaction effects on CRP in the UKB, stratified by which of the A compartment clusters the SNP lands in. Confidence intervals (dashed lines) were calculated for the A compartment cluster 3. The overall *p* value corresponds to the Kruskal–Wallis test for differences among all cluster *p* value distributions. Cluster 1 has a higher accumulation of low *p* value SNPs than cluster 5 in the post hoc Dunn test (*p* = 0.041 after correcting for multiple testing using the Holm procedure). CRP indicates C-reactive protein. Source data are provided as a Source Data file.

To determine whether any of the four main A compartment clusters are relatively more important in participating in the PAd genomic responses to BMI in the MZ twin pairs, we tested whether the A compartments with altered co-accessibility (Supplementary Data 4) are overrepresented in any of the clusters. We found that both clusters 1 (2.65-fold enrichment, *p*_adj_ = 2.62 × 10^−07^) and 2 (3.47-fold enrichment, *p*_adj_ = 2.81 × 10^−13^) are significantly enriched for the A compartments with altered co-accessibility (Supplementary Data 4). Interestingly, cluster 1 is significantly enriched for the heritability of CRP (Fig. 4a), BMI, TGs, ALT, blood glucose levels, systolic BP, and FVC (enrichment = 1.75-3.14; *p*_adj_ < 0.05). We did not observe a significant heritability enrichment of LDL and total cholesterol in the A compartment cluster 1, although the heritability of ALT, total cholesterol, systolic BP, and FVC was enriched in the A compartment cluster 2 (enrichment = 1.92-2.19; *p*_adj_ < 0.05). As a comparison with a non-cardiometabolic trait, the neuroticism score showed no significant heritability enrichment in any of the A compartment clusters. Strikingly, when we compare the GxE SNP *p* values for SNPs interacting with BMI to affect CRP levels in the UKB, we also found that cluster 1 has a higher accumulation of low *p* value GxE SNPs when compared to cluster 5 (*p*_KW_ = 0.0164) (Fig. 4b). This supports the conclusion that primed (cluster 1), rather than highly regulated and cell-type-specific regions (cluster 5), are important for the immunomodulatory effects of PAd responses to BMI. In summary, the cluster-dependent responses to BMI, contribution to the heritability of cardiometabolic traits, and GxEs affecting CRP in the UKB, all support a role for the A compartment cluster 1 region being important for PAd responses to BMI and affecting systemic inflammation and other obesity-related traits.

### Identification of BMI-responsive genes in the reprogrammed cluster 1 A compartments

We next searched for candidate BMI-responsive genes in the reprogrammed PAd A compartment cluster 1 regions. First, to identify genes with a high likelihood of being disrupted by reprogramming, we referred to our WGCNA co-expression modules (Supplementary Data 6). We use the module colors arbitrarily assigned by WGCNA to describe our gene co-expression module analyses to avoid confusion of module numbers with the A compartment cluster numbers. Two modules were enriched for A compartment cluster 1 genes: 472 of the 1054 black module genes (44.8%, 1.3-fold enrichment, *p*_adj_ = 3.01 × 10^−15^); and 116 of the 228 purple module genes (50.9%; 1.53-fold enrichment, *p*_adj_ = 2.92 × 10^−06^) are located within the cluster 1 A compartments (Supplementary Data 6 and Supplementary Table 6). We found that the first principal component (PC) of the black co-expression module is associated with the BMI status in the twins, both for the entire module (*n* = 1054 genes; *p*_adj_ = 5.5 × 10^−03^) as well as for the module genes that land within the A compartment cluster 1 regions (*n* = 472; *p*_adj_ = 3.1 × 10^−03^) (Supplementary Fig. 10; see Methods). None of the other PAd co-expression modules were associated with the BMI status in the twins (Supplementary Fig. 10). These results are in line with the black co-expression module being important in the MZ twin BMI discordance, and in particular, provide further support for the A compartment cluster 1 genes playing an important role in responding to BMI. Indeed, when we perform a KEGG pathway enrichment analysis on the cluster 1 genes in the black module, we find that they are enriched (enrichment ratio = 3.5; FDR <0.05) for genes in the parathyroid hormone synthesis, secretion, and action pathway (see Methods; Supplementary Data 7), which has previously been shown to be an axis through which adipose tissue is remodeled in obesity^39^.

Considering the black module association with BMI status in the twins, we posited that genes that are correlated with the first PC (PC1) of the module gene expression represent strong candidates for differential expression (DE) between the lower and higher BMI siblings. We, therefore, tested the 380 black module genes correlated with module PC1 (FDR <0.05) for DE (Supplementary Data 7). Given the small number of twin pairs, we applied an FDR <0.1 for the subsequent individual gene- and peak-level analyses. We found that 213 of the 380 genes (56.1%) are DE between the BMI status in the MZ twins, 52 of which are located in the reprogrammed A compartments (Supplementary Data 7).

Next, when searching the *cis* regions of the 52 DE genes for genetic enrichment of BMI GWAS variants utilizing the MAGENTA tool^40^ (see Methods), we observed a significant enrichment of BMI GWAS variants (*p* = 1.00 × 10^−04^) using the background set of genes as all A compartment cluster 1 genes in the black module. Notably, the enrichment of BMI GWAS SNPs remains significant even if only using a background of the remaining DE genes not in the reprogrammed A compartments (*p* < 0.05), highlighting the importance of these reprogrammed compartments in contributing to BMI. We further performed a TF motif enrichment analysis using HOMER on the ATAC-seq peaks in the surrounding regions of the 52 genes (gene ±250 kb), using the remaining black module genes in the A compartment cluster 1 as background. This identified four enriched motifs, including KLF7, which has previously been implicated in adipogenesis^41,42^, obesity and glucose-related pathological mechanisms^43,44^, and inflammatory signaling pathways in adipose tissue^43,45^ (Supplementary Table 7). In all, our results support the importance of the 52 DE genes in the reprogrammed A compartments as BMI response genes that ultimately may function as modulators of obesity-related traits.

### *INPP5K-MIR22HG* ATAC-seq peak co-accessibility is disrupted by increased BMI in the MZ siblings

To identify individual genomic regulatory elements that may differ between the lower and higher BMI MZ siblings, we first determined which ATAC-seq peaks are correlated with the expression of the 52 DE genes. We identified 143 preadipocyte ATAC-seq peaks correlated with the expression of 21 of the DE genes in the specific chromatin co-accessibility regions disrupted by increased BMI. These DE gene expression-correlated peaks land within seven of the 18 reprogrammed A compartments that contain DE genes, with most peaks (77; 53.8%) correlated with one gene (range 1–6 genes) (Supplementary Data 8).

**Fig. 5:**
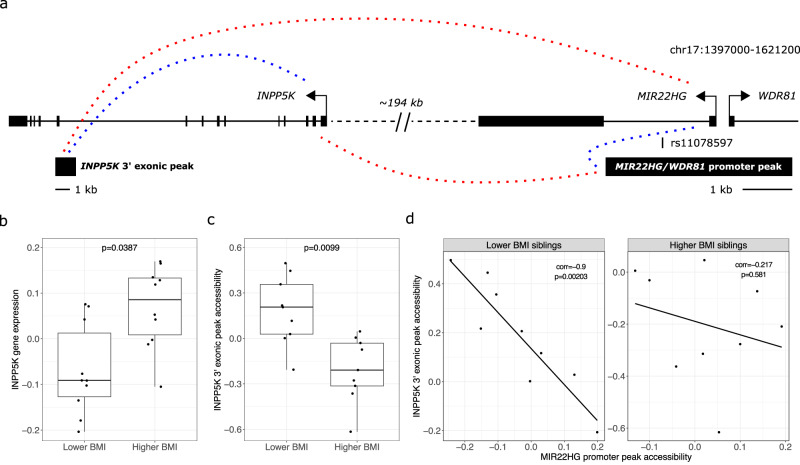
An example of a disruption in ATAC peak co-accessibility in the higher BMI MZ siblings and how it is linked to the twins’ differential expression of *INPP5K*. **a** Schematic of the genomic locus containing the *MIR22HG* promoter ATAC-seq peak that harbors a GWAS SNP rs11078597 for CRP, TGs, and ALT. Dashed lines represent significant correlations between peak accessibility and gene expression. Red dashed lines indicate positive correlations and blue dashed lines indicate negative correlations. Boxplots show the **b** differential expression of *INPP5K* (*n* = 10 pairs of MZ twins) and **c** differential accessibility of an ATAC-seq peak toward the 3’ end of the *INPP5K* gene (*n* = 9 pairs of MZ twins) between the lower and higher BMI MZ siblings’ preadipocyte data. *P* values correspond to the two-sided paired *t*-test. The boxplot center represents the median expression of the indicated gene set in the indicated compartment type, the upper and lower bounds of the box represent the 75th and 25th percentile, respectively, and the upper and lower whiskers represent the highest (non-outlier) and lowest (non-outlier) values, respectively. **d** The Spearman’s rank correlation between the *MIR22HG* promoter peak and the *INPP5K* exonic peak is shown for the lower (left, *n* = 9 lower BMI MZ siblings) and higher (right, *n* = 9 higher BMI MZ siblings) BMI MZ siblings separately. The significance of the difference in correlation was assessed using Fisher’s z-transformation (*p* = 0.027). Source data are provided as a Source Data file.

Next, we searched the GWAS catalog^46^ for SNPs that are associated with obesity-related traits and land in the DE gene-correlated ATAC peaks (Supplementary Table 8). Five of the seven reprogrammed A compartments containing peak-gene associations had GWAS SNPs in at least one of the expression-associated peaks, and one A compartment on chromosome 17 contains 12 (ten independent signals) of the total 19 (17 independent signals) cardiometabolic trait GWAS SNPs that land in the DE gene -correlated ATAC peaks (Supplementary Table 8). In particular, one of the SNPs within the promoters of the *MIR22HG*/*WDR81* genes (rs11078597) is associated with CRP, TGs, and ALT (Fig. 5a and Supplementary Table 8). This promoter peak is correlated with a distally located DE gene in the A compartment, inositol polyphosphate-5-phosphatase K (*INPP5K*) (Fig. 5a, b), but not the DE gene *WDR81* (Fig. 5a; Supplementary Data 8; and Supplementary Table 8). The peak is also correlated in the opposite direction with the long non-coding RNA (lncRNA) gene *MIR22HG* (Pearson’s *r* = −0.93, *p* = 2.75 × 10^−08^) (Fig. 5a), which is not DE in the twins but its expression is inversely correlated with *INPP5K* expression (Pearson’s *r* = −0.77, *p* = 1.71 × 10^-04^). These results motivated us to look further into *INPP5K* as an important DE gene responding to BMI in preadipocytes.

We searched for evidence of altered local *INPP5K* regulation between the lower and higher BMI MZ siblings by selecting the *MIR22HG* promoter peak containing the GWAS SNP rs11078597, as well as the ATAC-seq peaks within the *INPP5K* gene (±10 kb surrounding the gene region) for further follow-up (Fig. 5a and Supplementary Data 8). Five of the seven peaks within these regions are correlated with *INPP5K* expression, including a peak overlapping an exon near the 3’ end of the *INPP5K* gene that is inversely correlated with *INPP5K* expression (Fig. 5a and Supplementary Data 8). This exonic peak is the only one among the five *INPP5K* expression-correlated ATAC peaks that is inversely correlated with *INPP5K* expression, and it is differentially accessible (DA) between the lower and higher BMI MZ siblings (*p*_adj_ < 0.05) (Fig. 5c). The directly opposing gene expression correlations of the *INPP5K* exonic peak and the *MIR22HG* promoter peak (Fig. 5a) led us to hypothesize that these two ATAC peaks would be inversely correlated. Further, we hypothesized that this correlation (or co-accessibility) may be dependent on the BMI status in the MZ twin pairs, since *INPP5K* is DE and its exonic peak is DA between the higher and lower BMI MZ siblings (Fig. 5b). We, therefore, tested whether the correlation of the two peaks is different between the lower and higher BMI MZ siblings. We found that there is a strong inverse correlation of the *MIR22HG* promoter peak and the *INPP5K* exonic peak in the lower BMI MZ siblings that is not seen in the higher BMI siblings (*p* value for comparing the correlation coefficients using Fisher’s z-transformation = 0.027; see Methods) (Fig. 5d). These results demonstrate the regional co-accessibility disruption in higher BMI twin siblings and how it may relate to the DE of *INPP5K*. Based on GTEx data, human subcutaneous adipose tissue is the fourth most expressing tissue of *INPP5K* (median TPM 51.71 in 663 GTEx subcutaneous adipose samples) among the 54 tissues tested by GTEx, confirming that *INPP5K* is expressed in the human subcutaneous adipose tissue. Noteworthy, previous studies in animal models have shown that homozygous disruption of *Inpp5k* leads to embryonic lethality, while heterozygous mutant mice demonstrate normal food intake and adiposity but exhibit increased insulin sensitivity and reduced diet-induced obesity^47^. Combined with our result that *INPP5K* expression is upregulated in the higher BMI MZ siblings, this suggests that the regulatory circuit we highlight here (Fig. 5) is a BMI-responsive pathological mechanism contributing to obesity-related traits.

## Discussion

Obesity predisposes to COVID-19 complications and a cascade of cardiometabolic disorders (CMDs), likely at least partially by inducing chronic low-grade inflammation in the affected tissues^1,4–6,48^. Preadipocytes (PAd) are one of the key cell types in adipose tissue, responding to environmental cues and deciding whether to proceed toward fat storage (differentiation into adipocytes) or alternative pathways. In this study, we showed that increased BMI may affect the higher-order compartmentalization of the genome in PAd, in regions that contribute to the heritability of inflammation, measured by CRP in the UK Biobank (UKB). Furthermore, these genomic regions with altered co-accessibility in PAd that span a total of ~88.5 Mb exhibit a higher accumulation of small-effect GxBMI SNPs affecting CRP. Taken together, BMI seems to affect PAd co-accessibility in large genomic regions that contribute to systemic inflammation in humans, suggesting an important role for this progenitor cell type in the low-grade inflammatory state that is associated with obesity^49^.

PAd are an important cell type to consider when understanding systemic inflammation and downstream obesity comorbidities. However, PAd are an understudied cell type, underrepresented in large collections of epigenetic data such as ENCODE^50^ and Roadmap^51^, and mainly studied in mouse or human PAd-like cells, rather than primary cells^52,53^. This is likely in part due to the relative difficulty in collecting and propagating primary PAd cells in sufficient numbers for genomics studies. Because of this, there is an overall lack of knowledge surrounding the epigenomics of primary human PAd. Our study identified and characterized primary PAd A/B compartments, which represent the higher-order genomic compartmentalization of chromatin activity across BMI-discordant MZ twin pairs. Thus, this work advances the field by elucidating the genomic context in which local epigenetic signatures function, toward understanding how these cells integrate information from the environment to make important cellular decisions. We showed that the active PAd A compartments are enriched for enhancer and promoter chromatin states, relative to the more inactive B compartments, which are enriched for quiescent chromatin states. Furthermore, subclustering of the A compartments groups genomic regions that contain genes that are important for distinct progenitor cell functions, such as development (cluster 1), signal transduction (cluster 2), and hormone secretion (cluster 5). These clusters exhibit differences in their gene regulatory landscapes, assessed through chromatin state coverage, super-enhancer identification, regulatory interactions, and responses to differentiation signals. Thus, we have shown that higher-order genomic coordination is important for defining functionally related regions of the genome in PAd. By quantifying this coordination through the A compartment co-accessibility, we were then able to show that the PAd genomic programming at this level is impaired in the higher BMI siblings of the BMI-discordant MZ pairs.

We showed that the A compartments with altered co-accessibility contribute significantly to the heritability of CRP, meaning that environment-responsive regions of the genome also contain an enrichment of marginal SNP effects on inflammation. This suggests an important contribution of PAd BMI-responsive regions to systemic inflammation. GxEs are difficult to detect in humans for various reasons, including environmental heterogeneity, imprecision in the environmental measurements, and low power to detect interaction effects in the current cohort sizes, even in the UKB. We have previously shown that by restricting the GxE search space to regions of the genome that contain SNPs that respond to environmental cues in experiments, we can reduce the multiple testing burden to only include those regions with prior evidence of being relevant for that environmental stimulus^54^. This enables the detection of significant GxEs, even when genome-wide significant signals remain difficult to detect. Here, rather than a controlled experiment, we are using BMI as the environmental variable, which in itself is very heterogeneous in its etiology. By leveraging the BMI-discordant MZ twin pairs, we identified regions that differ between siblings, who exhibit decreased heterogeneity of genetic backgrounds that complicates and reduces power in environmental studies from cohorts of unrelated individuals. However, we recognize that the BMI-discordant MZ twin pairs may have been exposed to different environmental factors, ranging from increased caloric intake and decreased energy expenditure to other obesogenic and metabolic disruptors, such as drinking and smoking, medications, or food additives^55^, which each would warrant additional future studies with larger sample sizes.

We found that the regions with altered co-accessibility, marginal effects on CRP and other obesity-related traits, and GxE signals affecting CRP were enriched in regions of the genome that exhibit features of a more developmentally primed cellular state (cluster 1), rather than in the regions enriched for super-enhancers and highly interacting gene promoters (cluster 5). Cluster 1 exhibits a higher accumulation of promoter chromatin states, which possibly represent poised promoters. This is supported by the lower number of interactions per promoter, the lower gene expression, and the previous knowledge that promoter-promoter interactions are associated with a primed cellular state^31,56^. However, there are some limitations regarding this conclusion. It is important to consider that this conclusion may be driven by the fact that we mainly assessed the effects of BMI on the higher-order level of genome organization. The complex etiology of increased BMI reduces the power to detect BMI-driven differences in epigenetic signatures, particularly with the small sample size of the current study. This precluded the assessment of the effects of BMI on individual open chromatin peaks at the genome-wide level, due to the heavy multiple-testing burden at the genome-wide level (testing for BMI-driven differences in tens to hundreds of thousands of individual peaks). Therefore, it is possible that enhancer-enriched regions in PAd do respond to BMI, but the higher-order coordination of those regions, as we assessed through the PAd A compartment co-accessibility, is not as strongly affected. Of note, we did identify individual genes associated with the BMI differences between the MZ siblings, which was made possible by restricting our search space to the A compartments in cluster 1 that are likely to harbor differences between the BMI-discordant MZ twin pairs. This identified 52 DE genes in the reprogrammed cluster 1 A compartments, 21 of which exhibit correlations between their expression and local ATAC-seq peak accessibility. These 21 genes offer new candidates for future functional studies elucidating direct BMI effects on PAd biology, such as genetically modified PAd, to understand the function of these genes in this understudied cell type. Overall, to address the limitations in testing for DE genes and differentially accessible ATAC-seq peaks at the genome-wide level, larger cohort sizes and investigation of alternative genomic regulatory mechanisms aside from the higher-order coordination of active regions is warranted to further understand the effects of increased BMI on human primary PAd.

While we performed our study on primary PAd, it is important to note that the cells were cultured for a limited time (at most five passages) in the same media across MZ siblings and twin pairs. We find it interesting that the molecular alterations we see at the co-accessibility and gene expression level are maintained in vitro, but it is important to note that we do not have a direct comparison of PAd ex vivo (i.e., without culture), and thus our results as they pertain to the in vivo behavior of PAd must be interpreted with caution. Furthermore, it is important to consider the contribution of visceral adipose tissue preadipocytes to systemic inflammation. It has been suggested that visceral adipose tissue is even more prone to a pro-inflammatory profile than subcutaneous adipose tissue^57^. However, it is also postulated that efficient adipogenesis to expand subcutaneous adipose tissue is critical for buffering against lipotoxicity, and thus subcutaneous preadipocytes are likely relevant for visceral adipose tissue inflammation as well^58^.

In conclusion, we have characterized the higher-order genomic programming of human primary PAd and refined active genomic regions to functionally related clusters that span 30–130 Mbs, thus providing new important information for adipose biology and obesity research. Increased BMI seems to affect this level of PAd genomic programming through dysregulation of the coordination of functionally related regions of the genome. Our integrative analyses suggest that these regions are biologically meaningful for inflammation in humans by extending the results to an independent cohort, the UKB. The regions with altered co-accessibility are significantly enriched for the heritability of CRP, and harbor a higher accumulation of small-effect SNPs interacting with BMI to affect CRP levels. Taken together, our results suggest that the identified PAd-origin genomic regulatory mechanisms respond to BMI to induce the key obesity consequence, inflammation.

## Methods

### Study cohorts

Work in this manuscript is part of an ongoing study of BMI-discordant (ΔBMI ≥3 kg/m^2^) MZ twin pairs identified from the population-based Finnish twin cohorts (FTC), collected and recruited in the University of Helsinki, Helsinki, Finland. The twin study design was approved by the Ethics Committee of the Helsinki University Central Hospital, Helsinki, Finland, and all participants gave written informed consent. The FTC has conducted multiple, longitudinal surveys of Finnish twins, both MZ and DZ, born before 1958, 1975–1979, and 1983–1987^59–61^. MZ twin pairs with a large intrapair difference in BMI in the absence of medication and serious comorbidities were identified and invited for detailed metabolic phenotyping as described earlier^18,19^. Using genome-wide SNP array data, monozygosity was confirmed using the plink^62^ --genome function, which resulted in IBS estimates of 1.0 for all MZ siblings; as well as the GATK Picard Tools v2.9.0 GenotypeConcordance function that resulted in all twin pairs exhibiting a genotype concordance of >99%. The phenotypic characteristics are shown in Supplementary Data 1. The mean age is 47 y (±2 y (s.e.)), and it comprises 22 pairs of males (42%) and 31 pairs of females (58%) (*n* = 53 pairs) at the University of Helsinki. We successfully cultured the preadipocytes (PAd) for a subset of ten pairs (*n* = 20). This subset has a mean age of 42 y (±4 y (s.e.)), and it comprises six pairs of males (60%) and four pairs of females (40%). All participants gave written informed consent, and the study protocol was approved by the local ethics committee. The PAd sample collection is described below. For the C-reactive protein (CRP) genotype-by-environment interaction (GxE) analysis, we used the UK Biobank (UKB) cohort (*n* = up to 372,652 non-related Europeans)^63^, under Application Number 33934. UK Biobank has approval from the North West Multi-centre Research Ethics Committee (MREC) as a Research Tissue Bank (RTB) approval. All participants gave informed consent. The population sample used in this study has a mean age of 57 y ± 8 y (s.d.) and comprises 54% males and 46% females. The details of the GxE analysis are described below.

### PAd collection and cell culture from the BMI-discordant MZ twins’ adipose biopsies

We isolated the PAd from the subcutaneous fat biopsy specimens of the twin pairs undergoing adipose biopsies. Briefly, the biopsy specimens were first treated with collagenase and then centrifuged to separate the adipocytes from the stromal vascular fraction (SVF) pellet. Next, the SVF pellet was suspended to PAd basal media with 5% fetal calf serum supplemented with 1% penicillin-streptomycin. Then, the SVF was filtered before plating to allow the PAd to adhere and propagate. Finally, the viable PAd were cryopreserved for the downstream ATAC-seq and RNA-seq experiments (see below). To optimally preserve the in vivo epigenetic characteristics of these primary human cells, we will use the earliest passages (no more than five) for all experiments.

For the experiments, cryopreserved cells (passages 3–4) were seeded into PromoCell PAd growth medium (PromoCell C-27410) with 1% Gibco Penicillin-Streptomycin (Thermo Fisher 15140122) and cultured according to PromoCell PAd culturing protocols. Cells were maintained in a monolayer culture at 37 °C and 5% CO_2_. The primary PAd were passaged once for plating after propagation, resulting in fewer than five passages before collection for the experiments. We grew cells to <90% confluency for the PAd sample collection. We grew the cells to 100% confluency to begin the differentiation of these cells, using a PAd differentiation medium (PromoCell C-27436) for the 24-h differentiated (D1) time point. We collected two replicates of the PAd (for the ATAC-seq and RNA-seq) and one replicate of the D1 cells (for ATAC-seq), per individual.

### Assay for transposase-accessible chromatin (ATAC) –sequencing and data processing

We performed the ATAC-seq protocol in the PAd (*n* = 20) and D1 (*n* = 20) cells from each individual, including isogenic biological replicates (i.e., separate cultures from the same individual) from two twin pairs (*n* = 4 PAd and *n* = 4 D1), for a total of 48 samples. We followed the omni-ATAC protocol^64^, beginning with the 30 min DNase I treatment (Worthington LS002007; 200 U/ml), and then trypsinized cells using the PromoCell detach kit (PromoCell C-41210), according to the manufacturer’s protocols. Cells were lysed in ice-cold ATAC resuspension buffer (RSB) (10 mM Tris-HCl pH 7.4; 10 mM NaCl; 3 mM MgCl_2_) with 0.01% Digitonin, 0.1% Tween-20, and 0.1% Igepal on ice for 3 min. The lysis was quenched with ice-cold ATAC-RSB with 0.1% Tween-20. Nuclei were pelleted by centrifuging at 500 rcf for 10 min at 4 °C and resuspended in the transposition master mix (per sample: 25 ul 2x Illumina tagment DNA (TD) buffer, 2.5 ul Illumina TDE1 transposase, 16.5 ul PBS, 0.5 ul 1% digitonin, 0.5 ul 10% Tween-20, 5 ul H_2_O). The tagmentation reaction was incubated at 37 °C for 30 min with mixing at 1000 RPM. We purified the DNA and Illumina adapters were added by PCR. Libraries were sequenced on the Illumina HiSeq 4000 to produce an average of 40,315,572 (±14,577,770) reads. All ATAC-seq data were generated all at once, without any batches.

We processed the sequencing reads and performed quality control (QC) using the ENCODE ATAC-seq Data Standards and Prototype Processing Pipeline. Briefly, we aligned reads to the human reference genome (1000 Genomes v37) using Bowtie2 v2.2.9^65^ (with parameters -k 4 -X 2000 --local), filtering out unpaired mapped reads and reads with MAPQ <30 (Samtools v1.15^66^) and duplicates (marked with Picard Tools v2.9.0). Only reads from the autosomes were retained for downstream analyses.

Forty out of the 48 samples were retained for downstream analyses. One pair (*n* = 2) of twins failed the differentiation step; one sample had poor tagmentation and did not exhibit the proper fragment size distribution; four samples did not pass library complexity thresholds as defined by the ENCODA ATAC-seq Data Standards; and one sample had too few sequencing reads. Seven of the eight isogenic biological replicates from the same individual were retained after QC. These samples were used to assess the reproducibility of the data: the uncorrected peak BPMs for the seven isogenic biological replicates were correlated at a mean Spearman’s rho of 0.96. For comparison, the inter-individual PAd samples were correlated at a mean Spearman’s rho of 0.87 and the inter-individual differentiating PAd samples were correlated at a mean Spearman’s rho of 0.83.

For the samples that passed the QC, we called consensus peaks on all samples combined, after removing one of each isogenic biological replicate from the same individual (*n*_final_ = 33). Peaks were called using MACS2^67^ v2.2.7.1 and peaks with an FDR <0.05 were retained. We filtered out peaks in blacklisted regions^68^ using bedtools v2.25.0^69^ intersect function with the -v parameter, and retained peaks with counts per million (cpm) mapped reads ≥1 in at least 10% of the samples. When comparing the peaks called on individual samples to the consensus peak set, 85–99% of the individual peaks were also called in the consensus peak set; and 53–83% of all consensus peaks were called in each individual peak set, indicating a high reproducibility of called peaks.

We corrected the log_2_-transformed peak bins per million mapped reads (BPMs) for family ID (as a random effect), age, sex, and a fraction of read in peaks (FRiP), using the lme4^70^ v1.1 R package (Supplementary Fig. 2a–d and Supplementary Fig. 9). There are no patterns in the molecular trait sample correlation plots that would suggest batch effects in our data. These corrected BPMs were used in all analyses using the peak accessibility.

### A/B compartment detection from ATAC-seq data

We performed the A/B compartment detection as described previously in ref. ^20^. Briefly, first, we binned the PAd ATAC-seq sequencing reads for all complete pairs of twin PAd samples that passed QC (*n* = 9 pairs, for a total of 18 PAd ATAC-seq samples) into 100-kb bins across the genome, using the bedtools v2.25.0^69^ makewindows function and Subread featureCounts v1.6.4^71^, except for reads landing in blacklisted regions^68^, which were removed with the bedtools subtract function. We called the A/B compartments on all samples together so that we could quantify significant differences between the consensus A compartments between the lower and higher BMI twin siblings. We calculated the BPMs and corrected the log_2_-transformed BPMs for family ID (as a random effect), age, sex, and FRiP, using the lme4^70^ v1.1 R package. (Supplementary Fig. 2e–h). There are no patterns in the molecular trait sample correlation plots that would suggest batch effects in our data. Next, we obtained Spearman’s rank correlation matrix of the bins to get the pairwise bin co-accessibility measures. To call the A/B compartments, we calculated the first eigenvector of the correlation matrix, by chromosome, using the nipals function in the mixOmics^72^ v6.10.9 R package. Since the sign of the eigenvector is arbitrary, we used the known fact that the B compartments are generally more correlated than A compartments^20^. Thus, we correlated the eigenvector with the level of correlation of the compartment (sum of the correlation coefficients with all other bins on the chromosome), and ensured that the positive values in the eigenvector are negatively correlated with the level of bin correlation to denote A compartments as positive an B compartments as negative, changing the sign of the eigenvector if necessary. Next, we smoothed the eigenvector using a simple moving average (movavg function in the pracma v2.4.2 R package) with a bin size of 3 and obtained the final set of A/B compartments.

To permute the compartment locations for assessing the enrichment of pCHi-C in A compartments, we used the bedtools v2.25.0^69^ shuffle command with the -noOverlapping option, the -chrom option to shuffle the compartments within the same chromosome and the -excl option to exclude blacklisted regions that were removed when identifying A/B compartments.

To calculate the chromatin state coverage in the A/B compartments, we downloaded the ChromHMM^22^ 25-state segmentation across 127 reference epigenomes from the Roadmap Epigenomics Project. We determined the compartment coverage for each subset of ChromHMM states (enhancers, promoters, quiescent, and active) using bedtools v2.25.0^69^ intersect function and dividing by the length of the compartment.

### PAd RNA-sequencing and data processing

We isolated and purified RNA from the PAd cells from the ten pairs of twins, resulting in a total of 20 samples. Cells were washed with PBS once before lysing with TriZol (Invitrogen 15596026) and purified using Direct-zol RNA Mini-Prep (Zymo Research R2061). Libraries were prepared using the Illumina TruSeq Stranded mRNA kit and sequenced on an Illumina HiSeq 4000 instrument for an average sequencing depth of 78 M reads (±28 M reads) per sample. All RNA-seq data were generated all at once, without any batches.

Reads were aligned to human reference genome (1000 Genomes v37) with STAR v2.7.0e^73^, using the 2-pass method and the following parameters: --outFilterMultimapNmax 1, --outFilterMismatchNmax 6, --alignIntronMin 20, --alignIntronMax 500000, --chimSegmentMin 15. The various technical factors were obtained from STAR v2.7.0e^73^ after sequence alignment (uniquely mapped reads) or from the Picard Tools v2.9.0 (option CollectRnaSeqMetrics). We only retained genes with ≥1 cpm mapped reads in at least 10% of the samples.

We corrected the log_2_-transformed gene TPMs for family ID (as a random effect), age, sex, and median 3’ bias, using the lme4^70^ v1.1 R package (Supplementary Fig. 5). There are no patterns in the molecular trait sample correlation plots that would suggest batch effects in our data. These corrected TPMs were used in all analyses using the gene expression.

### The A compartment co-accessibility analysis

To quantify the compartment co-accessibility, we first calculated the BPMs for the A compartments and corrected the log_2_-transformed BPMs for family ID (as a random effect), age, sex, and FRiP, using the lme4^70^ v1.1 R package. Next, we obtained Spearman’s rank correlation matrix of the bins to get the pairwise bin co-accessibility measures. The level of co-accessibility per compartment is calculated as the sum of the compartment adjacency with all other compartments genome-wide, divided by the total number of compartments. Adjacency is equal to 0 if the Spearman’s ρ < 0.6 and equal to 1 if the Spearman’s ρ ≥ 0.6.

To compute the differences in compartment co-accessibility between the lower and higher BMI siblings, we separated the MZ twin pairs into subgroups containing the lower BMI siblings (*n* = 9) and higher BMI siblings (*n* = 9). We then calculated the compartment co-accessibility in the two subgroups separately, and compared the differences in co-accessibility at the compartment level by subtracting the co-accessibility value in the higher BMI MZ sibling group from the co-accessibility value in the lower BMI MZ sibling group. We used the one-sample Wilcoxon rank-sum (mu = 0) test to compare the level of co-accessibility between the lower and higher BMI siblings at the genome-wide level.

To identify individual compartments that are significantly different between the lower and higher BMI subgroups of siblings, we permuted the BMI status (higher or lower) within each MZ twin pair and re-calculated the co-accessibility differences of the compartments for all permutation (2^9^ pairs = 512 permutations). Permutations were outlined using the permute v0.9 R package. For each permutation, we compared the difference in the level of co-accessibility for a given compartment between the groups and compared it with the true difference in co-accessibility between the lower and higher BMI groups of siblings. We calculated the number of permutations that exhibited a higher co-accessibility in the lower BMI compared to higher BMI groups of siblings than the true difference (one-sided), given our previous result that there is a shift toward higher co-accessibility in the lower BMI sibling group at the genome-wide level. Compartments with a permutation *p* value of <0.01 (*n* = 121, totaling 88.5 Mb) were defined to have altered co-accessibility in the BMI-discordant twins and selected for downstream analyses.

### Partitioned LD-score (LDSC) regression

We used the partitioned LDSC regression method^26,27^ v1.0.1 to estimate the heritability of C-reactive protein (CRP), serum triglycerides (TGs), alanine aminotransferase (ALT), blood glucose levels, LDL and total cholesterol, systolic blood pressure (BP), forced vital capacity (FVC), and neuroticism score explained by the A/B compartments (stratifying the A compartments into the ones with altered co-accessibility or unaltered co-accessibility); or partitioned across the A compartment clusters. We downloaded the summary statistics for the second round of the UKB GWAS performed by the Neale Group and colleagues (http://www.nealelab.is/uk-biobank/).

### GxE analysis in the UKB

We downloaded the imputed genotype data from the UKB cohort^63^. We selected unrelated individuals of European ancestry who had both body mass index (BMI) and CRP measurements collected. We performed inverse normal transformation of the CRP values and corrected for age, age^2^, sex, assessment center ID, array type, and the first 20 genetic principal components. The BMI values were centered and scaled.

For the gene–environment interaction (GxE) analysis, we filtered out SNPs with a minor allele frequency of <1% and genotyping missing rate of >5%. To test for the interaction between the SNP and BMI, we used plink^62^ v1.90b3.45 to test the effect of the SNP, BMI, and BMIxSNP in a linear model.

### A compartment dimensionality reduction and clustering

Using UMAP for dimensionality reduction prior to clustering, as opposed to pairwise correlations to create an adjacency matrix, has previously been shown to improve the detection of true genetic interactions^74^. To do this, we first binned the PAd ATAC-seq sequencing reads into the identified PAd A compartments, except for reads landing in blacklisted regions^68^. We calculated the bins per million mapped reads (BPMs) and corrected the log_2_-transformed BPMs for family ID (as a random effect), age, sex, and FRiP, using the lme4^70^ v1.1 R package. We performed dimensionality reduction and clustering following the previously published methodology^74^: we performed principal component analysis (PCA) on the corrected PAd BPMs using the prcomp function in R. We then performed an additional dimensionality reduction to 2 components using Uniform Manifold Approximation and Projection (UMAP)^30^ in the umap v0.2.7.0 R package, with n_neighbors set to 10 and min_dist set to 0.05. We obtained the 75 nearest neighbors based on the UMAP projections for each compartment, which corresponds to the mean number of compartments each compartment is correlated with in pairwise correlation analyses. This was done using the get.knn function in the FNN^75^ v1.1.3 R package. Louvain clustering was performed on the resulting adjacency matrix, using the iGraph^76^ v1.2.6 R package, to obtain the 10 A compartment clusters used for downstream analyses.

For assessing the statistical significance of the differences between the A compartment clusters, we used the Kruskal–Wallis test and applied the dunnTest function in the FSA^77^ v0.8.32 R package for the post hoc test to determine which pairwise comparisons are significant after correcting for multiple testing using the Holm procedure.

### Preadipocyte super-enhancer identification

We used the sratoolkit v2.10.8 to download the raw FASTQ ChIP-seq data for the H3K27ac histone mark and MED1 at the day 1 adipogenic time point^53^ from bone marrow-derived stromal stem cells (BM-hMSC-TERT4) from the GEO database (accession code GSE113253). We processed the ChIP-seq data according to the ENCODE ChIP-seq pipeline. Briefly, sequencing reads were aligned to the human reference genome (1000 Genomes v37) reference genome using Bowtie2 v2.2.9^65^ (with parameters -k 4 --local), filtering out unmapped reads and reads with MAPQ < 30 (Samtools v1.15^66^) and duplicates (marked with Picard Tools v2.9.0). Only reads from the autosomes were retained for downstream analyses.

Peaks were called on each biological replicate separately using MACS2^67^ v2.2.7.1 and then consensus peaks were called on both replicates together to run the irreproducible discovery rate (IDR v2.0.3 [https://github.com/nboley/idr/]) analysis to identify reproducible peaks across both replicates. Only MED1 peaks that overlapped with H3K27ac peaks were retained as the constituent peaks for downstream analyses to identify super-enhancers. The ROSE algorithm^78,79^ [https://bitbucket.org/young_computation/rose/src/master/] was used to call super-enhancers based on the MED1 ChIP-seq alignments.

### Gene ontology (GO) term enrichment in the A compartment clusters

We performed a gene ontology (GO) enrichment analysis on the compartment genes in each A compartment cluster separately. As performing enrichment analyses on genes selected from large genomic regions can lead to spurious enrichments due to clusters of gene families or genes with similar functions^32^, we used the Network Enrichment Analysis Tool (NEAT) v1.2.3^33^ R package. NEAT uses information about the relationship between genes (e.g., genes in the same co-expression network) to test for functional enrichment, thereby requiring additional information about the gene function in that cell-type, on top of simply the region of the genome in which it lands.

To provide the network information to NEAT, we used the weighted gene co-expression network analysis (WGCNA) v1.72^34^ R package, using the RNA-seq data from all of the A compartment genes together. We followed default WGCNA procedures, except that we used a soft power value of 12 and performed a signed analysis. Genes from each A compartment cluster were assigned a co-expression module, and this network information was provided to NEAT for the A compartment GO enrichment. We downloaded the GO slims from the PANTHER^80^ database. We used an alpha of 0.005 as the cutoff for GO term significance to correct for testing 10 A compartment clusters separately, and then thresholded the within-compartment *p* values using an FDR <0.05 as the significance cut-point.

To summarize the cluster GO terms based on semantic similarity, we used the online tool REVIGO^81^. We used the simRel method for clustering, and then quantified the number of GO terms that are listed under each indispensable GO term from the REVIGO output.

### Transcription factor motif enrichment

To identify TF motif enrichments in this study, we used the HOMER^36^ v4.11.1 software de novo motif identification and enrichment assessment. For the A compartment cluster HOMER analyses, we used all ATAC-seq peaks within the PAd promoter–enhancer interactions identified in the PAd pCHi-C, for each A compartment cluster separately. We only included promoter–enhancer interactions in which the two ends of the interaction land in the same A compartment. As background, we used all the ATAC-seq peaks landing in the promoter–enhancer interactions within all other A compartment clusters.

For the TF motif enrichment in the regions surrounding the 52 DE genes in the reprogrammed A compartment cluster 1 regions, we selected all ATAC-seq peaks in the gene bodies (±250 kb). As background, we used all ATAC-seq peaks in the gene bodies (±250 kb) of the A compartment cluster 1 gene in the black gene co-expression module.

### Identification of differentially accessible ATAC-seq peaks in the first 24 h of PAd differentiation

We performed the differential accessibility (DA) analysis between the PAd and D1 time points using the R package limma v3.34.9^82,83^ and the voom^84^ method. We used the duplicateCorrelation function in limma^85^ to account for the repeated measure from the same individual. To decrease confounding, we included age, sex, and fraction of reads in peaks (FRiP) as covariates in the model and the family ID as a blocking factor. We tested for DA between the PAd and D1 time points in the lower and higher BMI individuals separately. We used an FDR <0.05 cutoff to define significant DA peaks for these comparisons.

### WGCNA module follow-up analyses

Module enrichment in A compartment clusters: We tested whether the genes in the 14 identified WGCNA co-expression modules are enriched in any of the 10 A compartment clusters, using the hypergeometric enrichment test implemented in the phyper function in R. We corrected for the multiple testing of 140 tests using the Bonferroni adjustment.

Module association with BMI status in the MZ twin pairs: We tested the effect of the BMI status in the MZ twin pairs on the first PC of the WGCNA gene co-expression modules, while accounting for twin pair ID as a random effect.

KEGG pathway enrichment: We used the WebGestalt^86^ tool to test for enrichment of KEGG pathways in the A compartment cluster 1 region black co-expression module genes. As background, we used all genes expressed in the A compartments.

Gene expression correlation with the first PC of the module gene expression: We correlated the expression of all A compartment cluster 1 genes in the black co-expression module with the first PC of the expression of these genes (*n* = 472 genes). We used Spearman’s rank correlation and corrected for multiple testing using the Benjamini–Hochberg adjustment, taking genes with a *p*_adj_ < 0.05 as significant.

### Differential gene expression and peak accessibility between the BMI-discordant MZ twin siblings

We assessed whether the 380 A compartment cluster 1 genes that are correlated with the first PC of the black co-expression module are DE between the lower and higher BMI MZ siblings by using a paired *t*-test on the corrected TPMs. We used a *p*_adj_ < 0.1 as the significance threshold for DE genes.

We tested whether the five ATAC-seq peaks that land within the *INPP5K* gene (±10 kb) are DA by using a paired t-test on the corrected TPMs. Due to the small number of peaks tested, we corrected for multiple testing with the Bonferroni adjustment.

### BMI GWAS enrichment analysis of the 52 DE gene regions using MAGENTA

We used Meta-Analysis Gene-set Enrichment of variaNT Associations (MAGENTA) v2.4^40^ to assess the 52 DE genes for significant BMI GWAS enrichment using the second round BMI GWAS summary statistics of the UK Biobank cohort from the Neale Lab (http://www.nealelab.is/uk-biobank/) and generating the association scores for each gene based on the *p* values of all variants within a 500 kb upstream and downstream window. MAGENTA natively filters out genes within close proximity of one another in the input set, but we retained all 52 genes in our analyses due to our small gene set size. As background, we used either 1) all genes in the A compartment cluster 1 region that are in the black gene co-expression module; or 2) all DE genes (*n* = 161) in the non-reprogrammed A compartment cluster 1 regions that are in the black gene co-expression module.

### Differential correlation between the lower and higher BMI MZ siblings

For the *MIR22HG* promoter peak and the *INPP5K* 3’ exonic peak co-accessibility differences, we used Fisher’s *z*-transformation to compare the correlation coefficients between the lower and higher BMI MZ siblings, as implemented in the cocor^87^ v1.1 R package.

All analyses using R were performed in R v3.6.3.

### Reporting summary

Further information on research design is available in the Nature Portfolio Reporting Summary linked to this article.

## Supplementary information

Supplementary Information

Description of Additional Supplementary Files

Supplementary Data 1

Supplementary Data 2

Supplementary Data 3

Supplementary Data 5

Supplementary Data 4

Supplementary Data 6

Supplementary Data 7

Supplementary Data 8

Reporting Summary

## Data Availability

Both the raw counts and normalized counts in transcript per million (TPMs) of the RNA-seq and ATAC-seq data used in the analyses of this study are available at the GEO database under the accession number GSE235363, and source data are provided with this paper. The data that support the GxE findings in this manuscript were generated using the UK Biobank under the UK Biobank Application Number 33934. These data were available under restricted access for bona fide researchers through the application process. The round 2 UK Biobank GWAS summary statistics used in this study are publicly available [http://www.nealelab.is/uk-biobank/]. The human reference genome (1000 Genomes human_g1k_v37) used in this study is available at IGSR [https://www.internationalgenome.org/category/assembly/]. The PAd pCHi-C data are available at the GEO database under the accession number GSE183770. The ChIP-seq data for the H3K27ac histone mark and MED1 at the day 1 adipogenic time point from bone marrow-derived stromal stem cells (BM-hMSC-TERT4) are available at the GEO database under the accession number GSE113253. The ENCODE blacklist used in this study is available at the ENCODE portal under the accession number ENCFF001TD. All data supporting the findings described in this manuscript are available in the article and in the Supplementary Information and from the corresponding author upon request. Source data are provided with this paper.

## Source data

Source Data

## Code availability

No custom code was used, and all codes used for analyses in this study were unaltered from their publicly available sources, as outlined in the Methods.
