# Supplementary material for: Increased body mass index is linked to systemic inflammation through altered chromatin co-accessibility in human preadipocytes
Source: Nat Commun. 2023 Jul 14;14:4214. doi: 10.1038/s41467-023-39919-y (PMC10349101; doi:10.1038/s41467-023-39919-y)
Supplement: Supplementary file 1 — Supplementary Information [file 41467_2023_39919_MOESM1_ESM.pdf]

Supplementary Table 1. ATAC-seq processing and quality control (QC) summary.

QC metric	mean	sd	range
Mean insert size	195	18	137 - 227
Fraction of reads uniquely aligned	0.730	0.032	0.657 - 0.773
Fraction of reads that are duplicates	0.069	0.029	0.045 - 0.204
Fraction of reads aligned to Mt	0.006	0.004	0.001 - 0.018
Final read count	69,671,524	15,146,435	41,096,376 - 106,257,726
NRF	0.848	0.041	0.703 - 0.912
PBC1	0.877	0.032	0.743 - 0.92
PBC2	9.833	1.952	4.19 - 13.263
FRiP	0.597	0.114	0.358 - 0.746
Number of peaks called	126,697	13,797	96,917 - 155,012

Mt indicates mitochondria; NRF, non-redundant fraction; PBC, PCR bottlenecking coefficient; FRiP, fraction of reads in peaks; and sd, standard deviation. Only samples that passed QC (n=40; this includes 33 independent samples and 7 isogenic biological replicates from the same individual) are included in the summary. Among the failed samples, one pair (n=2) of twins failed the differentiation step; one sample had poor tagmentation and did not exhibit the proper fragment size distribution; 4 samples did not pass library complexity thresholds, as defined by the ENCODE ATAC-seq Data Standards; and one sample had too few sequencing reads. Source data are provided as a Source Data file.

Supplementary Table 2. RNA-seq processing and QC summary.

QC metric	mean	sd	range
RIN	9.98	0.041	9.9 - 10
Fraction of reads uniquely aligned	0.931	0.007	0.913 - 0.942
Number of reads correct strand	59,247,414	22,237,588	21,729,476 - 127,517,226
Fraction of reads that are coding	0.645	0.009	0.628 - 0.662
Fraction of reads that are UTR	0.3	0.006	0.286 - 0.308
Fraction of reads that are intronic	0.038	0.005	0.03 - 0.049
Fraction of reads that are intergenic	0.018	0.001	0.016 - 0.02
Median 5’ bias	0.114	0.021	0.071 - 0.147
Median 3’ bias	0.143	0.042	0.076 - 0.19
Median 5’ to 3’ bias	0.889	0.092	0.689 - 1.097

RIN indicates RNA integrity number; and UTR, untranslated region. Source data are provided as a Source Data file.

Supplementary Table 3. Promoter Capture Hi-C sequencing read processing metrics.

	Reads	Uniquely mapped and paired	Unique ditags*	On-target reads	Capture efficiency
**PAd rep1**	106,651,878	77,323,631	46,453,815	37,763,852	0.813
**PAd rep2**	91,792,535	68,885,423	39,354,816	32,404,037	0.823

PAd indicates preadipocytes; and * indicates the unique ditag reads that remain after deduplication and filtering for technical artifacts in HiCUP[1].

Supplementary Table 4. Related to Figure 3. The A compartment cluster numbers and Mb spanned.

A compartment cluster	# of A compartments in cluster	Total Mb	# of A compartments with altered co-accessibility in cluster	Total Mb (altered co-accessibility)
1	145	129	30	37.3
2	144	88.8	39	32.5
3	107	42.6	13	5.0
4	156	52.8	8	1.8
5	138	60.6	11	4.0
6	137	39.3	1	0.1
7	193	78.1	7	4.0
8	121	27.1	6	1.1
9	180	69.2	4	2.4
10	230	62.3	2	0.3

Source data are from Supplementary Data 4.

Supplementary Table 5. Results from the *de novo* HOMER transcription factor motif enrichment analysis on the A compartment clusters.

A compartment cluster	TF motif	*p*-value
**1**	MEIS2	10^-24
**1**	ZNF416	10^-23
**1**	ZNF165	10^-22
**1**	ZNF460*	10^-21
**1**	Zbtb3*	10^-21
**1**	SREBF1	10^-19
**1**	Zbtb3*	10^-18
**1**	ZNF135	10^-17
**1**	YY2	10^-16
**1**	KLF3	10^-15
**1**	Sp4	10^-14
**1**	ZNF460*	10^-14
**1**	IRC900814	10^-14
**1**	YY1	10^-13
**1**	POU5F1	10^-13
**1**	PBX3	10^-13
**1**	Irf6	10^-13
**1**	GLI3	10^-12
**1**	Bcl11a	10^-12
**1**	PITX2	10^-12
**2**	WT1	10^-13
**3**	NR4A2	10^-13
**3**	MSANTD3	10^-13
**3**	MEIS1	10^-12
**3**	HNF4G	10^-12
**3**	NFATC4	10^-12
**5**	ZNF341	10^-17
**5**	Arid3a	10^-15
**5**	Gata5	10^-15
**5**	CHR	10^-14
**5**	Tcfap2a	10^-13
**5**	EGR2	10^-13
**5**	REL	10^-13
**5**	Six1*	10^-13

**5**	ZKSCAN5	10^-13
**5**	Six1*	10^-13
**5**	TBP	10^-12
**5**	Sox8	10^-12
**5**	Sox18	10^-12
**5**	ZNF768	10^-12
**5**	ZNF652	10^-12
**5**	HOXB4	10^-12
**7**	Pdx1	10^-12
**8**	ZNF415	10^-12
**10**	Mef2a	10^-20
**10**	ZNF135	10^-16
**10**	Irf4	10^-15
**10**	bZIP	10^-14
**10**	ZFP57	10^-13
**10**	Six1	10^-13
**10**	RUNX2	10^-12
**10**	ZBTB26	10^-12

ATAC-seq peaks in interacting promoters and enhancers within the indicated A compartment cluster were assessed for TF motif enrichment using HOMER[2], with all interacting peaks in other clusters as background. Asterisks * indicate motifs that were identified more than once in the *de novo* sequence identification. *P*-values correspond to the hypergeometric test for overrepresentation (one-sided) of the indicated motif in the indicated A compartment cluster peaks relative to the peaks in the background set (*i.e.*, all other clusters).

Supplementary Table 6. The WGCNA gene co-expression module enrichment in the A compartment cluster 1 regions.

WGCNA module	Enrichment	p_{hyper}	p_{adj}
turquoise	0.83	1.00E+00	1.00E+00
lightcyan	1.15	1.28E-01	1.00E+00
greenyellow	0.69	1.00E+00	1.00E+00
black	1.34	2.15E-17	3.01E-15
brown	0.99	6.53E-01	1.00E+00
blue	0.85	1.00E+00	1.00E+00
pink	0.93	8.10E-01	1.00E+00
purple	1.53	2.08E-08	2.92E-06
red	1.14	3.03E-02	1.00E+00
magenta	0.68	1.00E+00	1.00E+00
salmon	0.86	9.19E-01	1.00E+00
grey60	0.40	1.00E+00	1.00E+00
tan	1.11	1.54E-01	1.00E+00
midnightblue	1.19	6.23E-02	1.00E+00

Genes in the indicated WGCNA[3] co-expression module were assessed for their enrichment (hypergeometric test for overrepresentation (one-sided) and adjusted for multiple testing using the Bonferroni procedure) in the A compartment cluster 1 regions. The black and purple module genes are significantly enriched in these regions. The co-expression modules are randomly assigned color coding for the naming of the modules, and are directly from the WGCNA output. Source data are from Supplementary Table 5.

Supplementary Table 7. TF motif enrichment for the 52 DE genes in the reprogrammed A compartments.

De novo motif best TF match	Proportion of targets with motif	Proportion of background with motif	Enrichment ratio	P-value
MEF2C	0.0605	0.0235	2.6	1.00E-14
ZNF449	0.2679	0.1875	1.4	1.00E-13
KLF7	0.3092	0.2296	1.3	1.00E-12
ZBTB12	0.4754	0.3863	1.2	1.00E-12

ATAC-seq peaks in the 52 DE genes regions (gene body +/- 250 kb) were assessed for TF motif enrichment using HOMER[2], with all peaks in the A compartment cluster 1 black co-expression module genes (gene body +/- 250 kb) as background. *P*-values correspond to the hypergeometric test for overrepresentation (one-sided) of the indicated motif in the reprogrammed A compartment cluster 1 peaks relative to the non-reprogrammed A compartment cluster 1 peaks.

Supplementary Table 8. Cardiometabolic trait GWAS SNPs (all passing genome-wide significance with *p*$<5x10^{-08}$) landing in the DE gene expression-correlated ATAC-seq peaks.

A compartment	Peak ID	SNP ID	CMD trait
chr12_A_comp30	Peak_41090	rs11170386	Diastolic.blood.pressure
chr12_A_comp30	Peak_41109	rs11170481	Lung.function.(FVC)
chr17_A_comp12	Peak_72867	rs777657075	Waist-hip.index
chr17_A_comp12	Peak_72867	rs777657075	Waist-to-hip.ratio.adjusted.for.BMI
chr17_A_comp12	Peak_72897	rs2297508	Type.2.diabetes
chr17_A_comp1	Peak_71319	rs141977343	Hip.index
chr17_A_comp1	Peak_71319	rs2295480	Hip.index
chr17_A_comp1	Peak_71422	rs34582191	Lung.function.(FVC)
chr17_A_comp1	Peak_71447	rs11078597	C-reactive.protein
chr17_A_comp1	Peak_71447	rs11078597	Liver.enzyme.levels.(alkaline.phosphatase)
chr17_A_comp1	Peak_71447	rs11078597	Triglyceride.levels
chr17_A_comp1	Peak_71448	rs58360980	Waist.circumference.adjusted.for.body.mass.index
chr17_A_comp1	Peak_71491	rs112963849	Hypertension
chr17_A_comp1	Peak_71492	rs3803809	Lung.function.(FVC)
chr17_A_comp1	Peak_71492	rs4790310*	Apolipoprotein.A1.levels
chr17_A_comp1	Peak_71492	rs4790310*	HDL.cholesterol
chr17_A_comp1	Peak_71497	rs3760230*	Apolipoprotein.A1.levels
chr17_A_comp1	Peak_71497	rs3760230*	Nonalcoholic.fatty.liver.disease.(imputed)
chr17_A_comp1	Peak_71511	rs2281727±	Body.mass.index
chr17_A_comp1	Peak_71513	rs7217226±	Body.mass.index
chr17_A_comp1	Peak_71513	rs7217226±	Waist-hip.ratio
chr17_A_comp1	Peak_71538	rs57513571	Appendicular.lean.mass
chr6_A_comp26	Peak_155187	rs386906	Appendicular.lean.mass
chr7_A_comp52	Peak_170800	chr7:100295908	Type.2.diabetes
chr7_A_comp52	Peak_170806	rs2405442	Lung.function.(FEV1/FVC)

Only associations reaching genome-wide significance in the GWAS catalog[4] are listed. * indicates variants that are in linkage disequilibrium (LD) (r^2=0.93); ± indicates variants that are in LD (r^2=0.92).

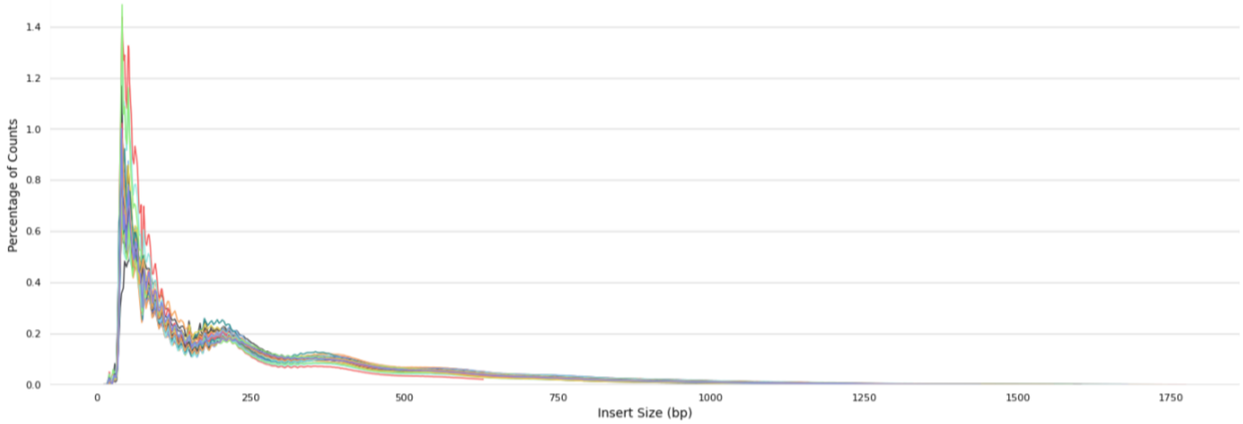

Supplementary Figure 1. ATAC-seq insert size distribution.

The distribution of the ATAC-seq library fragment insert sizes (x-axis) for all 40 samples that passed QC (see Supplementary Table 1; Methods), presented as the proportion of reads (y-axis) in each sample library containing the indicated fragment size. The periodicity corresponds to nucleosome-free regions (peak between 0-100 bp), mononucleosome regions (peak between 150-250 bp), dinucleosome regions (peak between 300-400 bp), and so on. Plot is the output from MultiQC[5]. Source data are provided as a Source Data file.

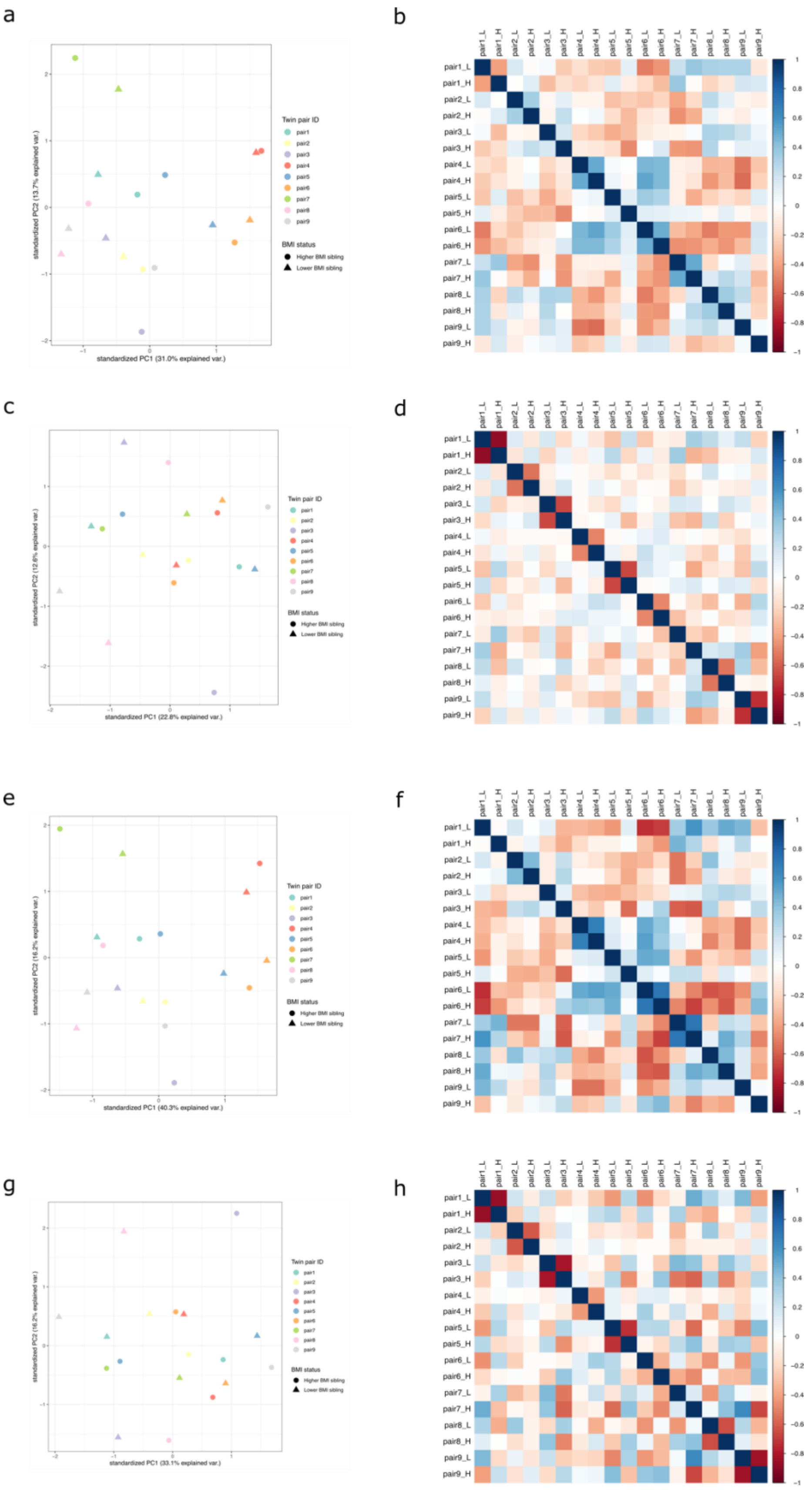

Supplementary Figure 2. Related to Figure 1.

Preadipocyte (PAd) ATAC-seq sample PCA and pairwise correlations on individual peak TPMs (a-d) or 100-kb bin TPMs (e-h). TPMs were first adjusted for sex, age, and fraction of reads in peaks (FRiP) to show that these BMI-discordant MZ twin pairs generally cluster near each other in the PCA plot (a, e) and exhibit positive correlations with each other (b, f), although this is not the case for all pairs. TPMs were adjusted for the family ID as a random effect, sex, age and fraction of reads in peaks (FRiP) (see Methods) and the resulting PCA plot (c, g) and correlation plot (d, h) are shown, wherein clustering is no longer twin pair ID -dependent. L indicates the lower BMI sibling within the MZ twin pairs; and H indicates the higher BMI sibling within the MZ twin pairs. Source data are provided as a Source Data file.

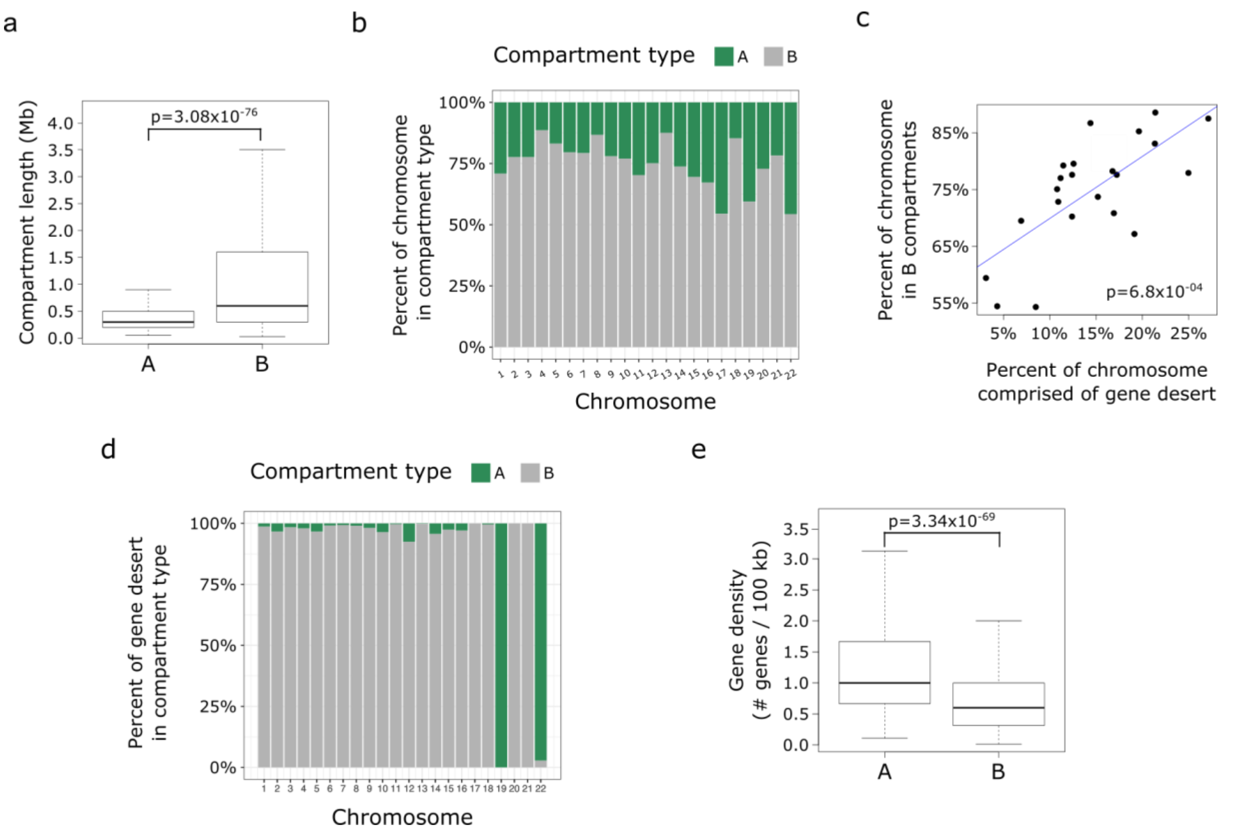

Supplementary Figure 3. Related to Figure 1. A/B compartment detection in preadipocytes reflects known genomic hallmarks of A and B compartments.

(a) Boxplots show the distribution of A/B compartment lengths. Outliers have been removed for clarity. The *p*-value corresponds to the two-sided Wilcoxon Rank Sum test comparing the lengths between the A (n=1,551 compartments) and B (n=1,557 compartments) compartments. (b) Bar plots show the proportion of each chromosome that makes up A/B compartments. (c) Scatterplot shows the relationship between the proportion of chromosomes made up of gene deserts, and the proportion of chromosomes made up of B compartments. The *p*-value corresponds to the significance of the Spearman's rank correlation coefficient. (d) Bar plots show the percent of gene deserts that overlap A or B compartments. (e) Boxplots show the gene density distribution in A/B compartments. Outliers have been removed for clarity. The *p*-value corresponds to the two-sided Wilcoxon Rank Sum test comparing the gene density between the A (n=1,278 compartments with at least one gene) and B (n=1,399 compartments with at

least one gene) compartments. For all boxplots, the center represents the median gene density of the compartment type, the upper and lower bounds of the box represent the 75th and 25th percentile, respectively, and the upper and lower whiskers represent the highest (non-outlier) and lowest (non-outlier) values, respectively. Source data are provided as a Source Data file.

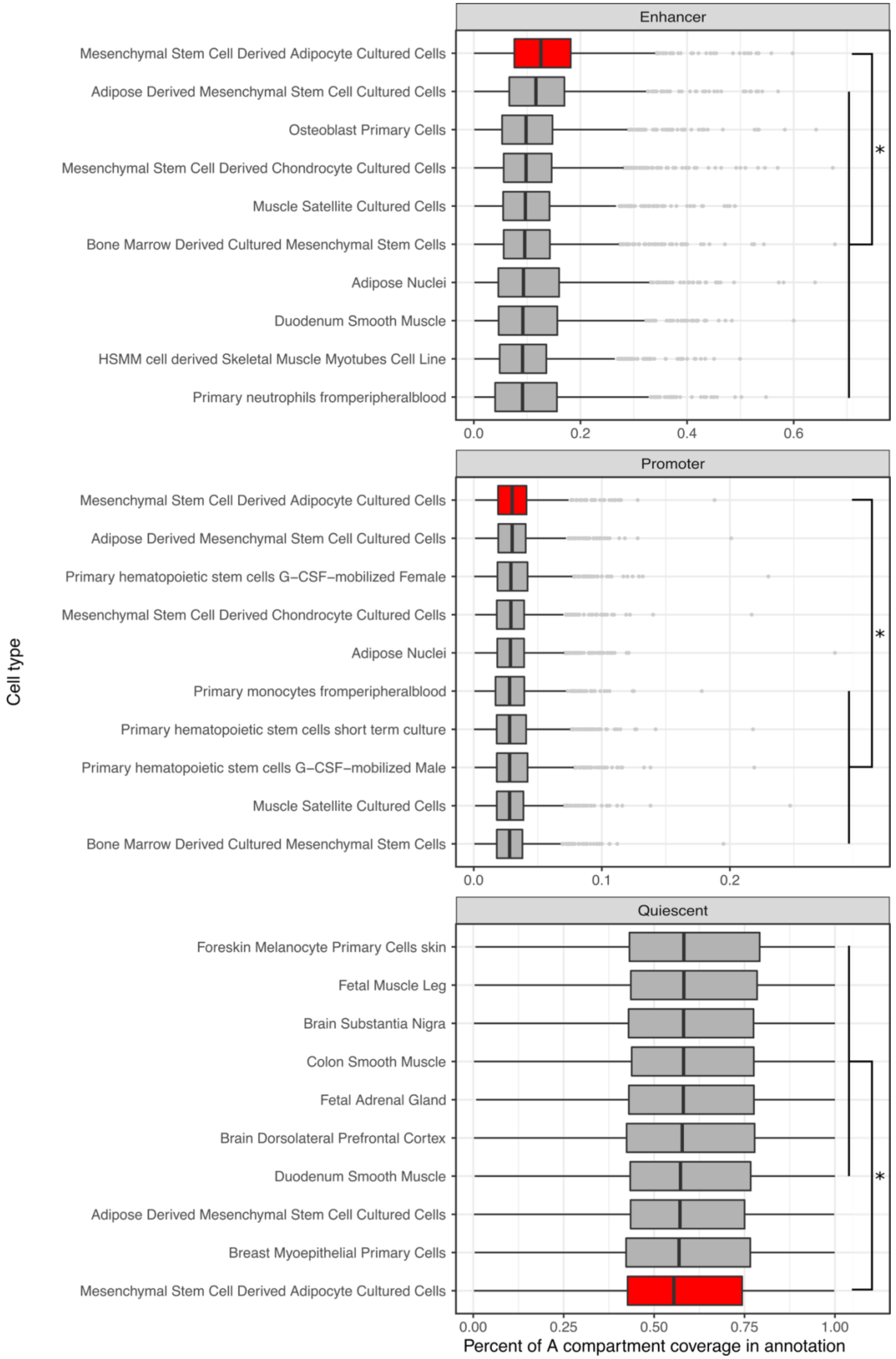
Enhancer
Mesenchymal Stem Cell Derived Adipocyte Cultured Cells
Adipose Derived Mesenchymal Stem Cell Cultured Cells
Osteoblast Primary Cells
Mesenchymal Stem Cell Derived Chondrocyte Cultured Cells
Muscle Satellite Cultured Cells
Bone Marrow Derived Cultured Mesenchymal Stem Cells
Adipose Nuclei
Duodenum Smooth Muscle
HSMM cell derived Skeletal Muscle Myotubes Cell Line
Primary neutrophils fromperipheralblood
0.0
0.2
0.4
0.6
*
Promoter
Mesenchymal Stem Cell Derived Adipocyte Cultured Cells
Adipose Derived Mesenchymal Stem Cell Cultured Cells
Primary hematopoietic stem cells G−CSF−mobilized Female
Mesenchymal Stem Cell Derived Chondrocyte Cultured Cells
Adipose Nuclei
Primary monocytes fromperipheralblood
Primary hematopoietic stem cells short term culture
Primary hematopoietic stem cells G−CSF−mobilized Male
Muscle Satellite Cultured Cells
Bone Marrow Derived Cultured Mesenchymal Stem Cells
0.0
0.1
0.2
*
Cell type
Quiescent
Foreskin Melanocyte Primary Cells skin
Fetal Muscle Leg
Brain Substantia Nigra
Colon Smooth Muscle
Fetal Adrenal Gland
Brain Dorsolateral Prefrontal Cortex
Duodenum Smooth Muscle
Adipose Derived Mesenchymal Stem Cell Cultured Cells
Breast Myoepithelial Primary Cells
Mesenchymal Stem Cell Derived Adipocyte Cultured Cells
0.00
0.25
0.50
0.75
1.00
*
Percent of A compartment coverage in annotation

Supplementary Figure 4. Related to Figure 1. The A compartment coverage of enhancer chromatin states across 127 ENCODE samples highlights the cell-type-specificity of the A compartment identification.

Boxplots show the coverage of enhancer, promoter, and quiescent ChromHMM[6] chromatin states across all of the A (n=1,551) compartments for the top 10 ENCODE[7] cell or tissue types (for the enhancer and promoter coverage); or the bottom 10 ENCODE cell or tissue types (for the quiescent coverage). The cell-type with the highest coverage of enhancer marks and promoter marks, as well as with the lowest coverage of quiescent marks, is denoted in red (mesenchymal stem cell -derived adipocyte cultured cells (MSC-Ad)). Asterisks correspond to $p<0.05$ the two-sided Wilcoxon Rank Sum test, comparing the MSC-Ad cells to all other cell- and tissue types, after correcting for multiple testing using the Benjamini-Hochberg procedure. Boxplot center represents the median coverage of the indicated chromatin state in the preadipocyte A compartments, the upper and lower bounds of the box represent the 75th and 25th percentile, respectively, and the upper and lower whiskers represent the highest (non-outlier) and lowest (non-outlier) values, respectively. Source data are provided as a Source Data file.

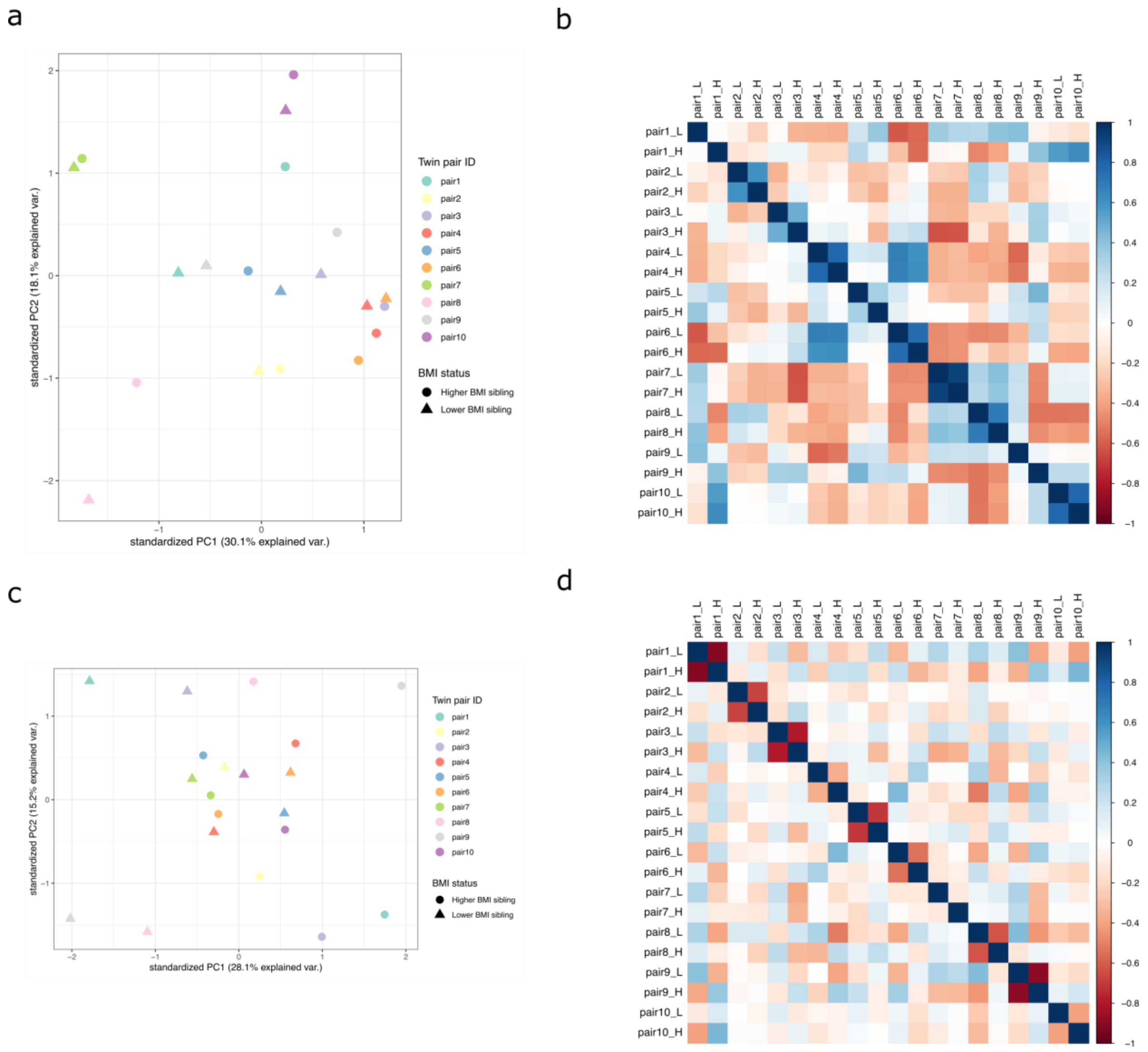

Supplementary Figure 5. Related to Figure 1.

RNA-seq sample PCA and pairwise correlations. Gene expression TPMs were first adjusted for sex, age, and median 3’ bias to show that these BMI-discordant MZ twin pairs generally cluster near each other in the PCA plot (a) and exhibit positive correlations with each other (b), although this is not the case for all pairs. Gene expression TPMs were adjusted for the family ID as a random effect, sex, age and and median 3’ bias (see Methods) and the resulting PCA plot (c) and correlation plot (d) are shown, wherein clustering is no longer twin pair ID -dependent. L indicates the lower BMI sibling within the MZ twin

pairs; and H indicates the higher BMI sibling within the MZ twin pairs. Source data are provided as a Source Data file.

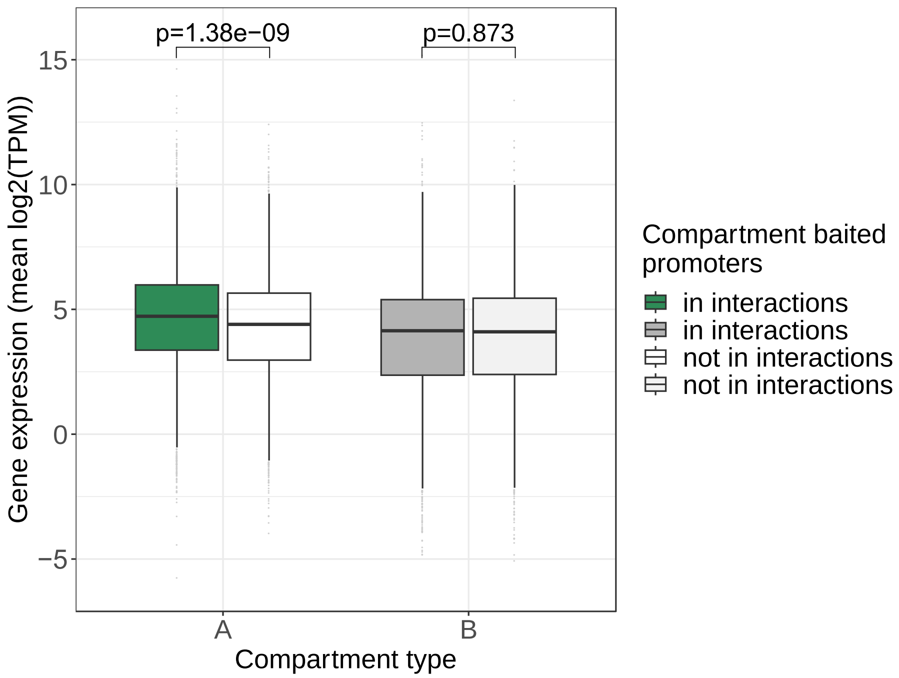

Supplementary Figure 6. Related to Figure 1. Promoter Capture Hi-C in PAd reveals how promoter interaction effects are dependent upon A/B compartmentalization.

Boxplot shows the expression of genes either involved in pCHi-C interactions (darker) or not (lighter) for genes with promoters in the A compartments (left; n=3,389 expressed genes in interactions; n=2,346 expressed genes not in interactions) or the B compartments (right; n=2,668 expressed genes in interactions; n=2,231 expressed genes not in interactions). *P*-value corresponds to the two-sided Wilcoxon Rank Sum test comparing gene expression within each compartment type separately. Boxplot center represents the median expression of the indicated gene set in the indicated compartment type, the upper and lower bounds of the box represent the 75th and 25th percentile, respectively, and the upper and lower whiskers represent the highest (non-outlier) and lowest (non-outlier) values, respectively. Source data are provided as a Source Data file.

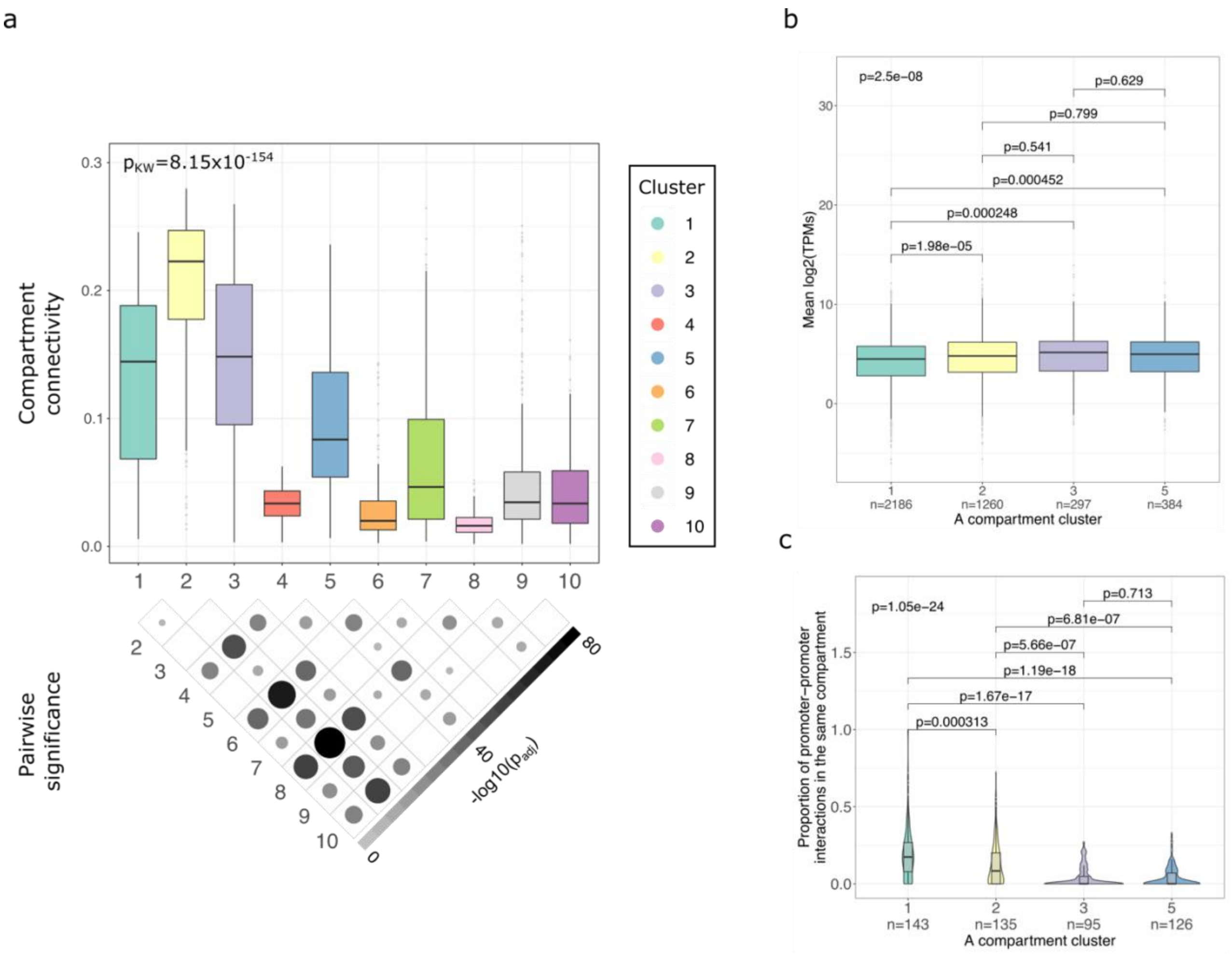

Supplementary Figure 7. Related to Figure 3. The A compartment clustering stratifies clusters based on the level of co-accessibility and cellular priming, assessed through promoter-promoter interactions and gene expression.

(a) Boxplots show the co-accessibility distributions across the 10 A compartment clusters. The overall *p*-value corresponds to the two-sided Kruskal-Wallis test and the *p*-value map below the plot denotes which pairwise differences are significant ($p_{adj}<0.05$) in the *post hoc* Dunn test, after correcting for multiple testing using the Holm procedure. The number of compartments in each cluster is listed in Supplementary Table 4. (b) Boxplots show the mean gene expression (log_2(TPM) of genes in each of the A compartment clusters. The overall *p*-value corresponds to the two-sided Kruskal-Wallis test, and the pairwise significance is determined by the *post hoc* Dunn test, after correcting for multiple testing using the Holm procedure. (c) Violin plots with inlaid boxplots show the proportion of pCHi-C interactions

within the same A compartment that are promoter-promoter interactions. The overall *p*-value corresponds to the two-sided Kruskal-Wallis test and the pairwise significance is determined by the *post hoc* Dunn test, after correcting for multiple testing using the Holm procedure. Violin plot shows the kernel probability density of the data. For all boxplots, the center represents the median gene density of the compartment type, the upper and lower bounds of the box represent the 75th and 25th percentile, respectively, and the upper and lower whiskers represent the highest (non-outlier) and lowest (non-outlier) values, respectively. Source data are provided as a Source Data file.

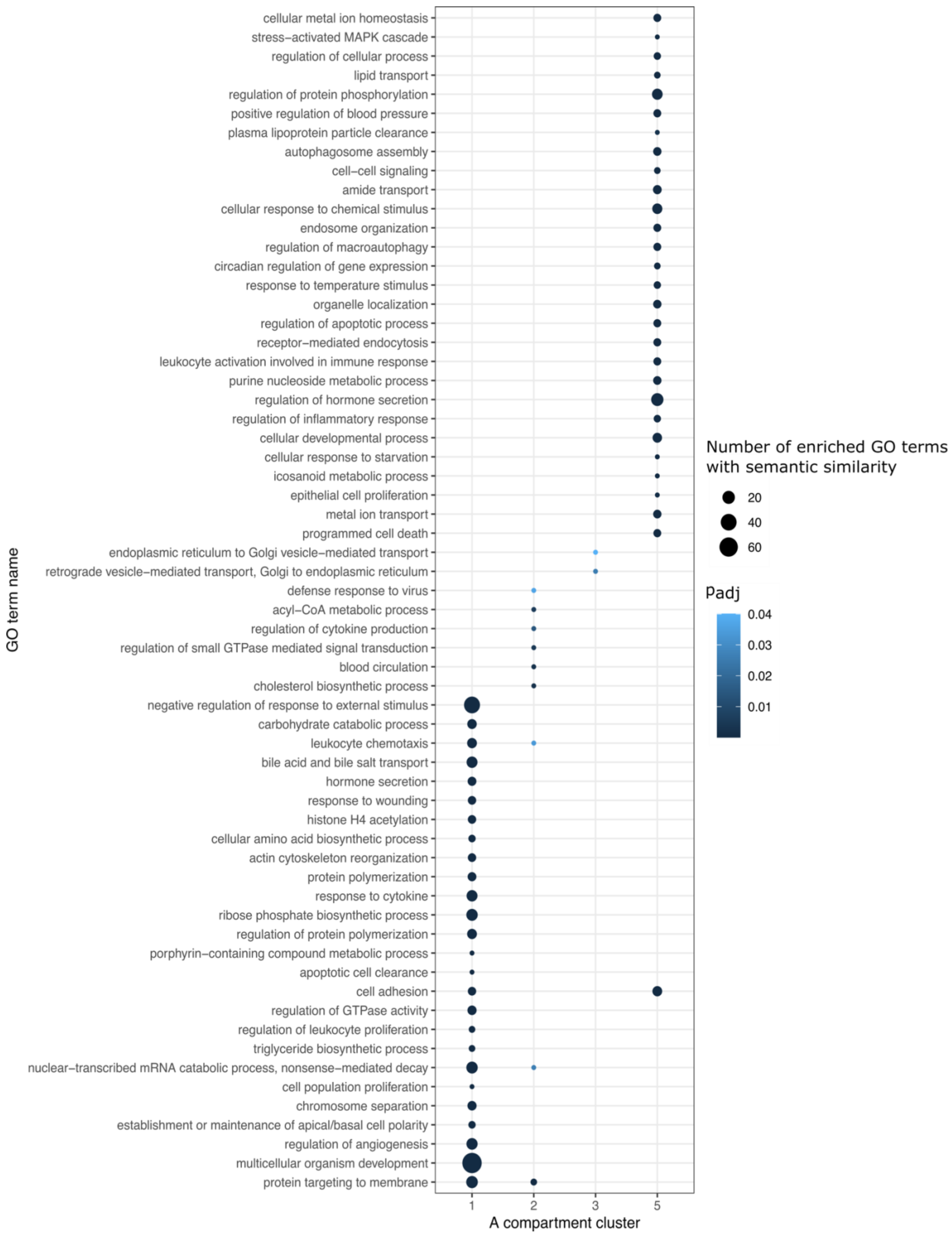

cellular metal ion homeostasis
stress−activated MAPK cascade
regulation of cellular process
lipid transport
regulation of protein phosphorylation
positive regulation of blood pressure
plasma lipoprotein particle clearance
autophagosome assembly
cell−cell signaling
amide transport
cellular response to chemical stimulus
endosome organization
regulation of macroautophagy
circadian regulation of gene expression
response to temperature stimulus
organelle localization
regulation of apoptotic process
receptor−mediated endocytosis
leukocyte activation involved in immune response
purine nucleoside metabolic process
regulation of hormone secretion
regulation of inflammatory response
cellular developmental process
cellular response to starvation
icosanoid metabolic process
epithelial cell proliferation
metal ion transport
programmed cell death
endoplasmic reticulum to Golgi vesicle−mediated transport
retrograde vesicle−mediated transport, Golgi to endoplasmic reticulum
defense response to virus
acyl−CoA metabolic process
regulation of cytokine production
regulation of small GTPase mediated signal transduction
blood circulation
cholesterol biosynthetic process
negative regulation of response to external stimulus
carbohydrate catabolic process
leukocyte chemotaxis
bile acid and bile salt transport
hormone secretion
response to wounding
histone H4 acetylation
cellular amino acid biosynthetic process
actin cytoskeleton reorganization
protein polymerization
response to cytokine
ribose phosphate biosynthetic process
regulation of protein polymerization
porphyrin−containing compound metabolic process
apoptotic cell clearance
cell adhesion
regulation of GTPase activity
regulation of leukocyte proliferation
triglyceride biosynthetic process
nuclear−transcribed mRNA catabolic process, nonsense−mediated decay
cell population proliferation
chromosome separation
establishment or maintenance of apical/basal cell polarity
regulation of angiogenesis
multicellular organism development
protein targeting to membrane
GO term name
1
2
3
5
A compartment cluster
Number of enriched GO terms with semantic similarity
20
40
60
Padj
0.04
0.03
0.02
0.01

Supplementary Figure 8. Related to Figure 3. Gene ontology (GO) term enrichment across the A compartment clusters.

Dot plot shows significantly enriched GO terms related to biological processes in the A compartment clusters 1, 2, 3, and 5. The size of the circle is proportional to how many of the significantly enriched GO terms for that A compartment cluster were listed together after semantic similarity analysis was done to cluster similar GO terms. The semantic similarity analysis was performed using REVIGO[8]. Source data are provided as a Source Data file.

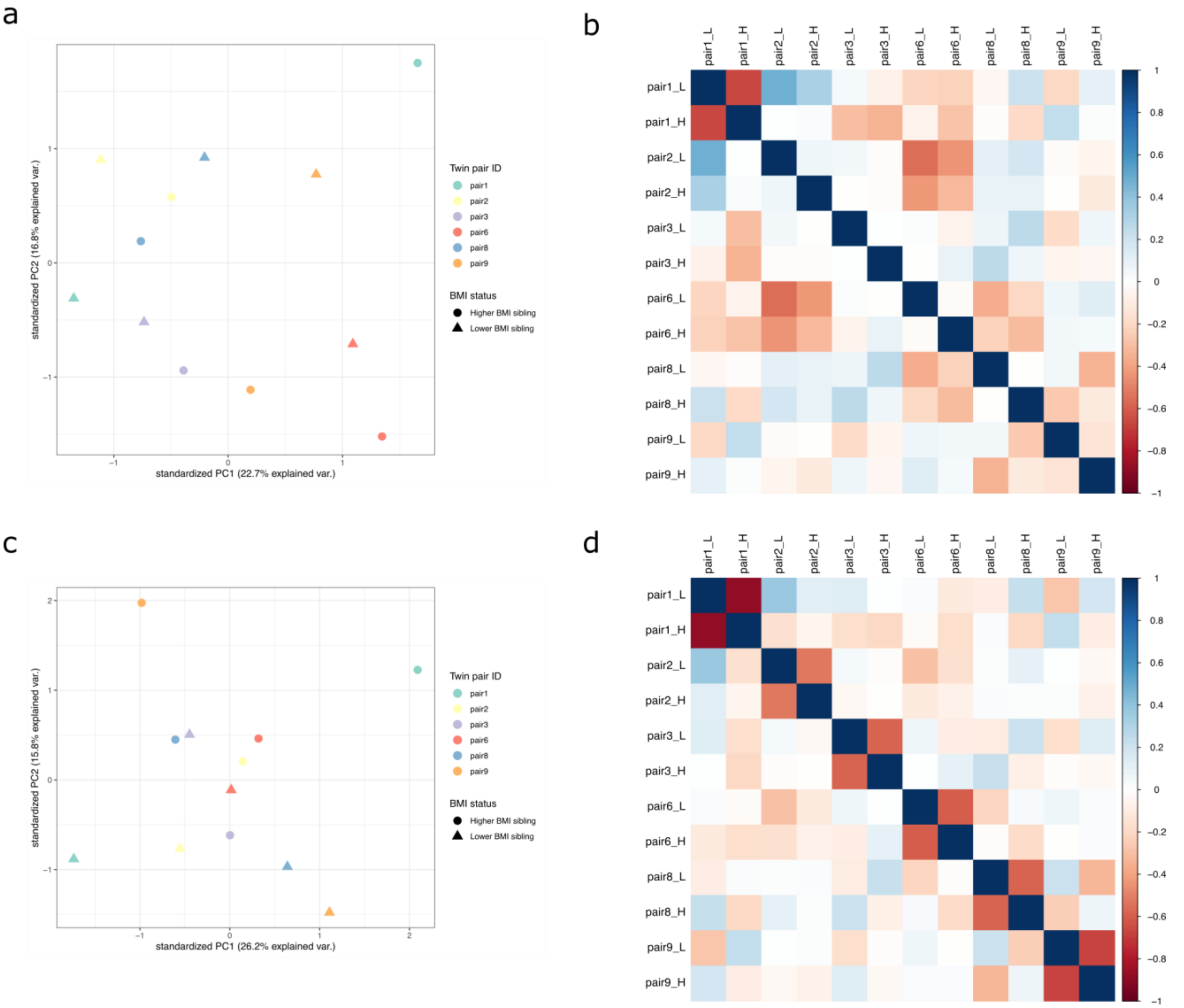

Supplementary Figure 9. Related to Figure 3.

Differentiating PAd ATAC-seq sample PCA and pairwise correlations on individual peak TPMs. TPMs were first adjusted for sex, age, and fraction of reads in peaks (FRiP); these BMI-discordant MZ twin pairs do not generally cluster near each other in the PCA plot (a) or exhibit strong positive correlations with each other (b). TPMs were adjusted for the family ID as a random effect, sex, age and fraction of reads in peaks (FRiP) (see Methods) and the resulting PCA plot (c) and correlation plot (d) are shown, wherein clustering is not twin pair ID -dependent. L indicates the lower BMI sibling within the MZ twin pairs; and H indicates the higher BMI sibling within the MZ twin pairs. Source data are provided as a Source Data file.

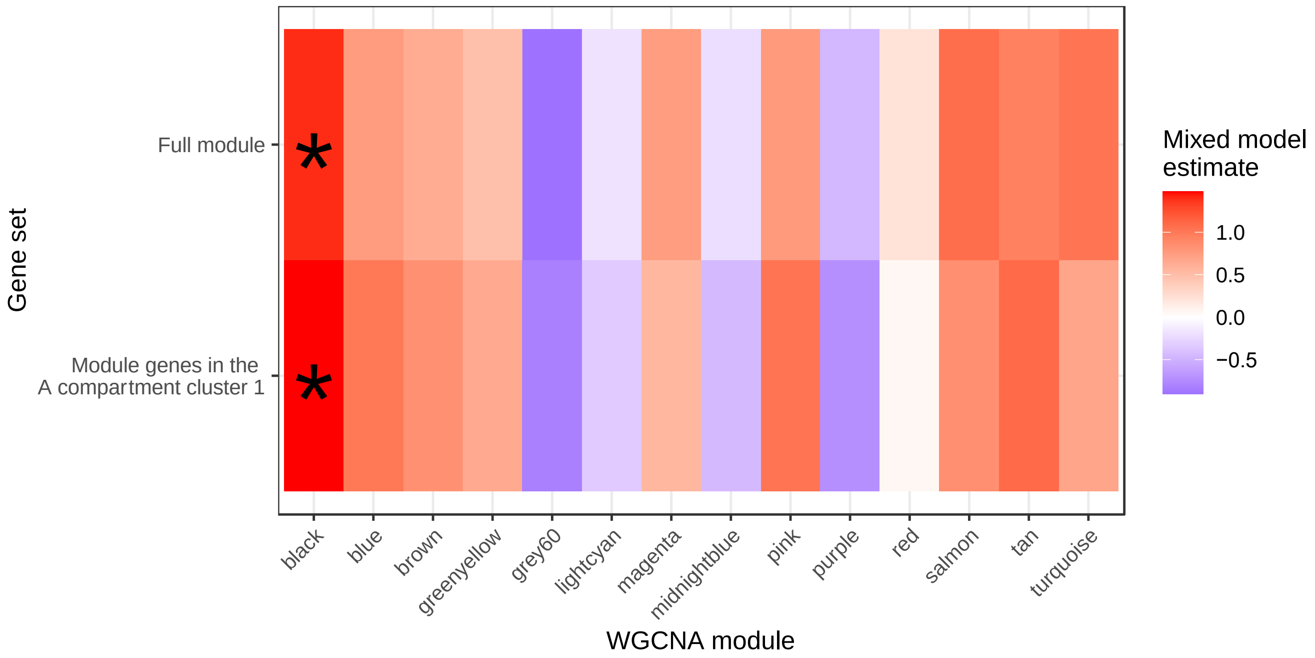

Supplementary Figure 10. Related to Figure 5. The black gene co-expression module is associated with the BMI status in the BMI-discordant MZ twin pairs.

Heatmap shows the estimates from a mixed model assessing the effect of the BMI status on the WGCNA[2] gene co-expression module eigengenes (first PC of the module gene expression), while controlling for the twin pair ID as a random effect (see Methods). Only the black gene co-expression module is associated with BMI status in the BMI-discordant MZ twin pairs after correction for multiple testing using the Benjamini-Hochberg procedure (*p_{adj}<0.01). The co-expression modules are randomly assigned color coding for naming the modules, and are directly from the WGCNA output. Source data are provided as a Source Data file.

Supplementary References

1. Wingett, S. W. *et al.* HiCUP: pipeline for mapping and processing Hi-C data. *F1000Research* **4**, 1310 (2015).

2 Heinz, S. *et al.* Simple combinations of lineage-determining transcription factors prime cis-regulatory elements required for macrophage and B cell identities. *Mol. Cell* **38**, 576–589 (2010).

3. Langfelder, P. & Horvath, S. WGCNA: An R package for weighted correlation network analysis. *BMC Bioinformatics* **9**, 559 (2008).

4. MacArthur, J. *et al.* The new NHGRI-EBI Catalog of published genome-wide association studies (GWAS Catalog). *Nucleic Acids Res.* **45**, D896–D901 (2017).

5. Ewels, P., Magnusson, M., Lundin, S. & Käller, M. MultiQC: Summarize analysis results for multiple tools and samples in a single report. *Bioinformatics* **32**, 3047–3048 (2016).

6. Ernst, J. & Kellis, M. ChromHMM: Automating chromatin-state discovery and characterization. *Nature Methods* vol. 9 (2012).

7. Davis, C. A. *et al.* The Encyclopedia of DNA elements (ENCODE): Data portal update. *Nucleic Acids Res.* **46**, D794–D801 (2018).

8. Supek, F., Bošnjak, M., Škunca, N. & Šmuc, T. Revigo summarizes and visualizes long lists of gene ontology terms. *PLoS One* **6**, e21800 (2011).
