# Supplementary material for: Increased body mass index is linked to systemic inflammation through altered chromatin co-accessibility in human preadipocytes
Source: Nat Commun. 2023 Jul 14;14:4214. doi: 10.1038/s41467-023-39919-y (PMC10349101; doi:10.1038/s41467-023-39919-y)
Supplement: Supplementary file 2 — Description of Additional Supplementary Files [file 41467_2023_39919_MOESM2_ESM.pdf]

Description of Additional Supplementary Information

Title: Supplementary Data 1. Metabolic trait measures by the lower and higher BMI sibling subgroups in the BMI-discordant MZ pairs.

Description: Not all pairs have measurements for all traits, and the number of pairs that went into each trait summary and statistical test (two-sided paired *t*-test) is indicated in the "npairs" column. Type 2 diabetes (T2D) status (known in 50 pairs) is not significantly different between the lower [n=4/50 (8.0%) have T2D] and higher [n=12/50 (24%) have T2D] BMI subgroups of siblings (p_{chisq}=0.056). Smoking increases inflammation; in this cohort the smoking status (known in 47 pairs) is not significantly different between the lower [n=11/47 (23%) are smokers] and higher [n=7/47 (15%) are smokers] BMI subgroups of siblings (p_{chisq}=0.43). While there are significant differences in liver enzymes (GGT), there are no significant differences in alcohol intake between the lower and higher BMI siblings (see Table). SE indicates standard error; BMI, body mass index; WHR, waist-to-hip ratio; HbA1c, hemoglobin A1c; HOMA-IR, homeostatic model assessment for insulin resistance; HDL, high-density lipoprotein; TG, triglycerides; LDL, low-density lipoprotein; CRP, C-reactive protein; AST, aspartate transaminase; ALT, alanine transaminase; and GGT, gamma-glutamyltransferase. For participant phenotypic data, aggregate level data are shown to safeguard the privacy of the individual participant's phenotypic data. Excluding the BMI grouping and our adjustments for age and sex, we are not using any of these phenotypic data in the actual analyses of our manuscript, rather the phenotypic information is only provided as a general descriptive information of the twin cohort.

Title: Supplementary Data 2. Metabolic trait measures in the ten BMI-discordant MZ pairs from whom we collected preadipocyte data in this study.

Description: Not all pairs have measurements for all traits, and the number of pairs that went into each trait summary and statistical test (two-sided paired *t*-test) is indicated in the "npairs" column. RNA-seq data collected from the preadipocytes from all 10 pairs passed QC. SE indicates standard error; BMI, body mass index; WHR, waist-to-hip ratio; HbA1c, hemoglobin A1c; HOMA-IR, homeostatic model assessment for insulin resistance; HDL, high-density lipoprotein; TG, triglycerides; LDL, low-density lipoprotein; CRP, C-reactive protein; AST, aspartate transaminase; ALT, alanine transaminase; and GGT, gamma-glutamyltransferase. For participant phenotypic data, aggregate level data are shown to safeguard the privacy of the individual participant's phenotypic data. Excluding the BMI grouping and our adjustments for age and sex, we are not using any of these phenotypic data in the actual analyses of our manuscript, rather the phenotypic information is only provided as a general descriptive information of the twin cohort.

Title: Supplementary Data 3. Metabolic trait measures in the nine MZ sibling pairs passing ATAC-seq quality control.

Description: Not all pairs have measurements for all traits, and the number of pairs that went into each trait summary and statistical test (two-sided paired *t*-test) is indicated in the "npairs" column. SE indicates standard error; BMI, body mass index; WHR, waist-to-hip ratio; HbA1c, hemoglobin A1c; HOMA-IR, homeostatic model assessment for insulin resistance; HDL, high-density lipoprotein; TG, triglycerides; LDL, low-density lipoprotein; CRP, C-reactive protein; AST, aspartate transaminase; ALT, alanine transaminase; and GGT, gamma-glutamyltransferase. For participant phenotypic data, aggregate level data are shown to safeguard the privacy of the individual participant's phenotypic data. Excluding the BMI grouping and our adjustments for age and sex, we are not using any of these phenotypic data in the actual analyses of our manuscript, rather the phenotypic information is only provided as a general descriptive information of the twin cohort.

Title: Supplementary Data 4. A/B compartment genomic coordinates.

Description: A/B compartment chromosomal locations. The A compartments are listed as either reprogrammed or non-reprogrammed (see Methods), and the A compartment cluster assignments are listed. Chr indicates chromosome.

Title: Supplementary Data 5. The A compartment cluster gene ontology enrichment results.

Description: The NEAT[1] results of the A compartment cluster gene ontology (GO) analysis (see Methods). Nab indicates number of links between the cluster 1 genes (a) and the GO term (b); and expected_nab indicates the expected number of links between a and b. *P*-values correspond to a two-sided hypergeometric test, and were adjusted for multiple testing using the FDR method.

Title: Supplementary Data 6. Preadipocyte WGCNA co-expression module assignment for A compartment genes.

Description: WGCNA[2] was performed on all A compartment genes (see Methods). The A compartment in which the gene lands is listed, along with the assigned compartment cluster. The co-expression module number and randomly assigned color coding for naming the modules is directly from the WGCNA output.

Title: Supplementary Data 7. The A compartment cluster 1 genes in the black co-expression module.

Description: The 472 genes in the A compartment cluster 1 regions and assigned to the black co-expression module are listed. The FDR cutoff for the correlation of the gene expression with the first PC of the black co-expression module (‘Black module PC1 cor FDR’ column) was 0.05. The FDR cutoff for

the differential expression between the lower and higher BMI twin siblings ('DE gene FDR' column) was 0.1.

Title: Supplementary Data 8. ATAC-seq peak correlations with the 52 black co-expression module DE genes in the reprogrammed cluster 1 A compartments.

Description: The 52 DE genes were tested for whether their expression is correlated with ATAC-seq peak accessibility for all ATAC-seq peaks within the same A compartment as the DE gene. Only significant correlations are reported (FDR<0.1). Asterisks * mark peaks that are correlated with our candidate gene *INPP5K* and land either in the *MIR22HG* promoter peak containing the GWAS SNP rs11078597 (Peak_71447) or in the *INPP5K* genes (+/- 10 kb). Only the Peak_71424 is inversely correlated with *INPP5K* expression.

Supplementary Data References

1. Signorelli, M., Vinciotti, V. & Wit, E. C. NEAT: An efficient network enrichment analysis test. *BMC Bioinformatics* **17**, 352 (2016).

2. Langfelder, P. & Horvath, S. WGCNA: An R package for weighted correlation network analysis. *BMC Bioinformatics* **9**, 559 (2008).
