# Supplementary material for: Increased body mass index is linked to systemic inflammation through altered chromatin co-accessibility in human preadipocytes
Source: Nat Commun. 2023 Jul 14;14:4214. doi: 10.1038/s41467-023-39919-y (PMC10349101; doi:10.1038/s41467-023-39919-y)
Supplement: Supplementary file 11 — Reporting Summary [file 41467_2023_39919_MOESM11_ESM.pdf]

nature portfolio

Corresponding author(s): Paivi Pajukanta

Last updated by author(s): Jun 28, 2023

Reporting Summary

Nature Portfolio wishes to improve the reproducibility of the work that we publish. This form provides structure for consistency and transparency in reporting. For further information on Nature Portfolio policies, see our Editorial Policies and the Editorial Policy Checklist.

Statistics

For all statistical analyses, confirm that the following items are present in the figure legend, table legend, main text, or Methods section.

n/a	Confirmed	
☐	☒	The exact sample size (n) for each experimental group/condition, given as a discrete number and unit of measurement
☐	☒	A statement on whether measurements were taken from distinct samples or whether the same sample was measured repeatedly
☐	☒	The statistical test(s) used AND whether they are one- or two-sided<br>*Only common tests should be described solely by name; describe more complex techniques in the Methods section.*
☐	☒	A description of all covariates tested
☐	☒	A description of any assumptions or corrections, such as tests of normality and adjustment for multiple comparisons
☐	☒	A full description of the statistical parameters including central tendency (e.g. means) or other basic estimates (e.g. regression coefficient) AND variation (e.g. standard deviation) or associated estimates of uncertainty (e.g. confidence intervals)
☐	☒	For null hypothesis testing, the test statistic (e.g. F, t, r) with confidence intervals, effect sizes, degrees of freedom and P value noted<br>*Give P values as exact values whenever suitable.*
☒	☐	For Bayesian analysis, information on the choice of priors and Markov chain Monte Carlo settings
☒	☐	For hierarchical and complex designs, identification of the appropriate level for tests and full reporting of outcomes
☐	☒	Estimates of effect sizes (e.g. Cohen's d, Pearson's r), indicating how they were calculated

Our web collection on statistics for biologists contains articles on many of the points above.

Software and code

Policy information about availability of computer code

Data collection	We used the sratoolkit v2.10.8 to download the raw H3K27ac and MED1 ChIP-seq data from GEO (accession code GSE113253).
Data analysis	As described in the methods, processing the BMI-discordant MZ twin ATAC-seq and RNA-seq sequencing data was performed as follows: ATAC-seq reads were aligned using Bowtie2 v2.2.9 and filtered using Samtools v1.15. Peaks were then called using MACS2 v2.2.7.1 and peaks in blacklisted regions were removed with the bedtools v2.25.0 intersect function using the -v parameter. RNA-seq reads were aligned using STAR v2.7.0e. Picard Tools v2.9.0 was used to collect RNA-seq technical metrics. The Subread featureCounts v1.6.4 was used to assign aligned sequencing reads to gene transcripts, ATAC-seq peaks, or 100-kb genomic bins (obtained using the bedtools v2.25.0 makewindows function). Blacklisted regions were removed from ATAC-seq 100-kb bin counts using the bedtools v2.25.0 subtract function. Bins per million mapped reads (BPMs) for the ATAC-seq peaks (and 100-kb bins for the A/B compartment calling) and transcripts per million mapped reads (TPMs) for the RNA-seq gene expression data were corrected for family ID (as a random effect), age, sex, and Fraction of Reads in Peaks (FRiP, ATAC-seq) or median 3' bias (RNA-seq), using the lme4 v1.1 R package.<br><br>Identification of A/B compartments was performed using the nipals function of the mixOmics v6.10.9 R package to obtain the first eigenvector of the Spearman's rank correlation matrix of the 100-kb bin ATAC-seq BPMs. A simple moving average with a bin size of 3 (using the movavg function in the pracma v2.4.2 R package) was used to obtain the final set of A/B compartments. To permute compartment locations for assessing promoter Capture Hi-C enrichment in the A compartments, we used the bedtools v2.25.0 shuffle function with the -noOverlapping and -chrom parameters, as well as the -excl parameter to exclude blacklisted regions. The ChromHMM chromatin state coverage was obtained using the bedtools v2.25.0 intersect function and dividing by the compartment length. To identify A compartments that differ in connectivity between the lower and higher BMI MZ siblings, we used the permute v0.9 R package to define all possible (2^9 pairs) 512 permutations.

Dimensionality reduction for A compartment clustering was performed first using the prcomp function in R, then using the UMAP v0.2.7.0 R package to perform dimensionality reduction to 2 components, and the k nearest neighbors were obtained using the FNN v1.1.3 R package. Final clustering was done with the Louvain algorithm implemented in the iGraph v1.2.6 R package. Comparing various metrics across the A compartment clusters was done using the Kruskal-Wallis test in R and then applying the post hoc Dunn test using the FSA v0.8.32 R package.

For super-enhancer identification, H3K27ac and MED1 ChIP-seq reads were aligned with Bowtie2 v2.2.9 and MACS2 v2.2.7.1 was used to call peaks. The publicly available IDR v2.0.3 (https://github.com/nboley/idr/) software was used to identify reproducible peaks across biological replicates. The publicly available ROSE algorithm (https://bitbucket.org/young_computation/rose/src/master/) was used to call super-enhancers.

The NEAT v1.2.3 R package was used to test for GO term enrichment in the A compartment clusters. To provide network information to NEAT, we used the WGCNA v1.72 R package. The online tool REVIGO was used to cluster GO terms based on semantic similarity. Transcription factor motif enrichment in the ATAC-seq peaks was performed using HOMER v4.11.1. KEGG pathway enrichment analysis was performed using the online tool WebGestalt.

Differential accessibility between PAd and differentiating PAd (D1) ATAC-seq data was done using the limma v3.34.9 R package. Differential correlation analysis was done using the cocor v1.1 R package.

To estimate partitioned heritability for BMI and obesity-related traits, partitioned LD score regression (LDSC v1.0.1) was used. For genotype-by-environment interaction testing, we used plink v1.90b3.45. The publicly available software MAGENTA v2.4 was used to test for regional GWAS SNP enrichments.

All other statistical analyses were performed using R v3.6.3.

For manuscripts utilizing custom algorithms or software that are central to the research but not yet described in published literature, software must be made available to editors and reviewers. We strongly encourage code deposition in a community repository (e.g. GitHub). See the Nature Portfolio guidelines for submitting code & software for further information.

Data

Policy information about availability of data

All manuscripts must include a data availability statement. This statement should provide the following information, where applicable:

- Accession codes, unique identifiers, or web links for publicly available datasets
- A description of any restrictions on data availability
- For clinical datasets or third party data, please ensure that the statement adheres to our policy

Both the raw counts and normalized counts in transcript per million (TPMs) of the RNA-seq and ATAC-seq data used in the analyses of this study are available at the GEO database under the accession number GSE235363 [https://www.ncbi.nlm.nih.gov/geo/query/acc.cgi?acc=GSE235363], and source data are provided with this paper. The data that support the GxE findings in this manuscript were generated using the UK Biobank under the UK Biobank Application Number 33934. These data are available under restricted access for bona fide researchers through the application process. The round 2 UK Biobank GWAS summary statistics used in this study are publicly available [http://www.nealelab.is/uk-biobank/]. The human reference genome (1000 Genomes human_g1k_v37) used in this study is available at IGSR [https://www.internationalgenome.org/category/assembly/]. The PAd pCHi-C data are available at the GEO database under the accession number GSE183770 [https://www.ncbi.nlm.nih.gov/geo/query/acc.cgi?acc=GSE183770]. The ChIP-seq data for the H3K27ac histone mark and MED1 at the day 1 adipogenic time point from bone marrow derived stromal stem cells (BM-hMSC-TERT4) are available at the GEO database under the accession number GSE113253 [https://www.ncbi.nlm.nih.gov/geo/query/acc.cgi?acc=GSE113253]. The ENCODE blacklist used in this study is available at the ENCODE portal under the accession number ENCFF001TD [https://www.encodeproject.org/annotations/ENCSR636HFF/]. All data supporting the findings described in this manuscript are available in the article and in the Supplementary Information and from the corresponding author upon request.

Research involving human participants, their data, or biological material

Policy information about studies with human participants or human data. See also policy information about sex, gender (identity/presentation), and sexual orientation and race, ethnicity and racism.

Reporting on sex and gender	We report the nearly balanced design between male and female sex in our BMI-discordant MZ twin study and correct for sex in our ATAC-seq and RNA-seq data. We did not perform sex-based analyses due to the limited sample size in the current study.
Reporting on race, ethnicity, or other socially relevant groupings	Only age and sex were controlled for in our analyses. No socially constructed or socially relevant categorization variables were used in our analyses.
Population characteristics	The BMI-discordant MZ twin pairs were identified from the population-based Finnish Twin Cohorts (FTC), and selected based on large intrapair differences in BMI (>=3 kg/m2). The mean age of the BMI-discordant pairs is 47 years (+/- 2y (s.e.)) and comprises 22 pairs of males (42%) and 31 pairs of females (58%). The subset of twins from the ongoing Finnish twin cohorts (FTC) for which we cultured and collected preadipocyte data has a mean age of 42 y (+/- 4 y (s.e.)), and it comprises 6 pairs of males (60%) and 4 pairs of females (40%).<br><br>The GxE research was conducted using the UK Biobank Resource. The UK Biobank consists of ~500,000 individuals with genotypes and phenotypes and includes males and females from a broad range of ages. The maximum number of individuals was included to increase the power of discover of GxE interactions (n=up to 372,652 non-related individuals). This population sample has a mean age of 57 y +/- 8 y (s.d.) and comprises 54% males and 46% females.
Recruitment	MZ twin pairs with large intrapair differences in BMI (>=3 kg/m2) were identified from the FTC and invited for detailed

	metabolic phenotyping. No evident self-selection biases are noted.
Ethics oversight	The MZ twin study was approved by the Ethics Committee of the Helsinki University Central Hospital, Helsinki, Finland and all participants gave informed consent. Participants received 75 Euros per day for 2 days for their participation in the full twin study, of which our study is a part. UK Biobank has approval from the North West Multi-centre Research Ethics Committee (MREC) as a Research Tissue Bank (RTB) approval. All UK Biobank participants gave informed consent.

Note that full information on the approval of the study protocol must also be provided in the manuscript.

Field-specific reporting

Please select the one below that is the best fit for your research. If you are not sure, read the appropriate sections before making your selection.

☒ Life sciences ☐ Behavioural & social sciences ☐ Ecological, evolutionary & environmental sciences

For a reference copy of the document with all sections, see nature.com/documents/nr-reporting-summary-flat.pdf

Life sciences study design

All studies must disclose on these points even when the disclosure is negative.

Sample size	For the ATAC-seq and RNA-seq data collection from the BMI-discordant monozygotic (MZ) twin pairs' preadipocytes (ATAC + RNA) and differentiating preadipocytes (day 1 of differentiation, ATAC only), sample size was determined by the successful culture of the preadipocytes for 10 pairs (n=20). The gene-environment interaction (GxE) analysis was conducted using the UK Biobank Resource. The UK Biobank consists of ~500,000 individuals with genotypes and phenotypes. The maximum number of individuals available was included to increase the power of discover of GxE interactions (n=up to 372,652 unrelated Europeans). The used sample sizes are typical for published functional genomics experiments in ENCODE and other functional databases, which are limited by the availability of human biopsies and primary cell biobanking. The functional priors established through our experiments helped identify regions with altered open chromatin co-accessibility harboring variants that contribute to systemic inflammation through their interactions with BMI on C-reactive protein (CRP) in the more powerful UK Biobank analyses, further supporting the adequate sample sizes of the primary cell data.
Data exclusions	For the ATAC-seq data, exclusion criteria related to data quality control (QC) were set ahead of time to correspond to ENCODE Data Standards. This resulted in the exclusion of 6 samples due to the following reasons: improper fragment size distribution (1); poor library complexity (4); and too few sequencing reads (1). To reduce spurious associations due to population substructure and genetic heterogeneity in the UK Biobank analysis, related and non-Caucasian individuals were excluded.
Replication	Two BMI-discordant MZ twin pairs were cultured in two independent cultures to serve as replication to test for the reproducibility of the ATAC-seq data. We collected PAd (n=4) and differentiating PAd (n=4) ATAC-seq data for these two pairs. Seven of the eight isogenic biological replicates from the same individual were retained after QC. These samples were used to assess the reproducibility of the data: the uncorrected peak TPMs for the seven isogenic biological replicates were correlated at a mean Spearman's rho of 0.96. For comparison, the inter-individual PAd samples were correlated at a mean Spearman's rho of 0.87 and the inter-individual differentiating PAd samples were correlated at a mean Spearman's rho of 0.83.<br><br>Results reported from the BMI-discordant MZ twin cohort were not replicated due to the unique nature of the cohort in identifying epigenetic differences across BMI levels within MZ pairs. Results reported from the UK Biobank GxE analyses were not replicated due to the unprecedented large sample size of the cohort and because individual GxE SNPs were not identified after correction for multiple testing.
Randomization	N/A. This is an observational study, so no randomization was performed.
Blinding	N/A. This is an observational study, so no randomization was performed.

Reporting for specific materials, systems and methods

We require information from authors about some types of materials, experimental systems and methods used in many studies. Here, indicate whether each material, system or method listed is relevant to your study. If you are not sure if a list item applies to your research, read the appropriate section before selecting a response.

Materials & experimental systems

n/a	Involved in the study
☒	☐ Antibodies
☐	☒ Eukaryotic cell lines
☒	☐ Palaeontology and archaeology
☒	☐ Animals and other organisms
☒	☐ Clinical data
☒	☐ Dual use research of concern
☒	☐ Plants

Methods

n/a	Involved in the study
☒	☐ ChIP-seq
☒	☐ Flow cytometry
☒	☐ MRI-based neuroimaging

Eukaryotic cell lines

Policy information about cell lines and Sex and Gender in Research

Cell line source(s)	ZenBio SP-F-2, lot L120116E
Authentication	The human primary preadipocytes were obtained from the established and reputable commercial vendor, ZenBio and passed standard viability and differentiation capacity requirements prior to being made available.
Mycoplasma contamination	The human primary preadipocytes were tested by ZenBio for common microbiological contaminants and infectious viruses, including Mycoplasma, and found to produce negative results prior to the cells being made available. As these are primary cells, they have not been passaged extensively, and are unlikely to survive Mycoplasma contamination. Furthermore, we observed no evidence of contamination during the culture period.
Commonly misidentified lines (See ICLAC register)	N/A
